# Neuregulin-1 controls an endogenous repair mechanism after spinal cord injury

**DOI:** 10.1093/brain/aww039

**Published:** 2016-03-17

**Authors:** Katalin Bartus, Jorge Galino, Nicholas D. James, Luis R. Hernandez-Miranda, John M. Dawes, Florence R. Fricker, Alistair N. Garratt, Stephen B. McMahon, Matt S. Ramer, Carmen Birchmeier, David L. H. Bennett, Elizabeth J. Bradbury

**Affiliations:** ^1^The Wolfson Centre for Age-Related Diseases, Regeneration Group, King’s College London, Guy’s Campus, London Bridge, London, UK; ^2^Nuffield Department of Clinical Neurosciences, West Wing, John Radcliffe Hospital, Oxford, UK; ^3^Max Delbrück Center for Molecular Medicine, Berlin, Germany; ^4^Charité Universitätsmedizin Berlin, Charitéplatz, Berlin, Germany; ^5^International Collaboration on Repair Discoveries, The University of British Columbia, Vancouver, Canada

**Keywords:** spinal cord injury, axon degeneration, remyelination, demyelination, transgenic model

## Abstract

Following traumatic spinal cord injury, acute demyelination of spinal axons is followed by a period of spontaneous remyelination. However, this endogenous repair response is suboptimal and may account for the persistently compromised function of surviving axons. Spontaneous remyelination is largely mediated by Schwann cells, where demyelinated central axons, particularly in the dorsal columns, become associated with peripheral myelin. The molecular control, functional role and origin of these central remyelinating Schwann cells is currently unknown. The growth factor neuregulin-1 (Nrg1, encoded by *NRG1*) is a key signalling factor controlling myelination in the peripheral nervous system, via signalling through ErbB tyrosine kinase receptors. Here we examined whether Nrg1 is required for Schwann cell-mediated remyelination of central dorsal column axons and whether Nrg1 ablation influences the degree of spontaneous remyelination and functional recovery following spinal cord injury. In contused adult mice with conditional ablation of Nrg1, we found an absence of Schwann cells within the spinal cord and profound demyelination of dorsal column axons. There was no compensatory increase in oligodendrocyte remyelination. Removal of peripheral input to the spinal cord and proliferation studies demonstrated that the majority of remyelinating Schwann cells originated within the injured spinal cord. We also examined the role of specific Nrg1 isoforms, using mutant mice in which only the immunoglobulin-containing isoforms of Nrg1 (types I and II) were conditionally ablated, leaving the type III Nrg1 intact. We found that the immunoglobulin Nrg1 isoforms were dispensable for Schwann cell-mediated remyelination of central axons after spinal cord injury. When functional effects were examined, both global Nrg1 and immunoglobulin-specific Nrg1 mutants demonstrated reduced spontaneous locomotor recovery compared to injured controls, although global Nrg1 mutants were more impaired in tests requiring co-ordination, balance and proprioception. Furthermore, electrophysiological assessments revealed severely impaired axonal conduction in the dorsal columns of global Nrg1 mutants (where Schwann cell-mediated remyelination is prevented), but not immunoglobulin-specific mutants (where Schwann cell-mediated remyelination remains intact), providing robust evidence that the profound demyelinating phenotype observed in the dorsal columns of Nrg1 mutant mice is related to conduction failure. Our data provide novel mechanistic insight into endogenous regenerative processes after spinal cord injury, demonstrating that Nrg1 signalling regulates central axon remyelination and functional repair and drives the trans-differentiation of central precursor cells into peripheral nervous system-like Schwann cells that remyelinate spinal axons after injury. Manipulation of the Nrg1 system could therefore be exploited to enhance spontaneous repair after spinal cord injury and other central nervous system disorders with a demyelinating pathology.

## Abstract (precis)

Spontaneous remyelination after spinal cord injury is mediated largely by Schwann cells of unknown origin. Bartus *et al*. show that neuregulin-1 promotes differentiation of spinal cord-resident precursor cells into PNS-like Schwann cells, which remyelinate central axons and promote functional recovery. Targeting the neuregulin-1 system could enhance endogenous regenerative processes.

## Graphical abstract

**Figure:**
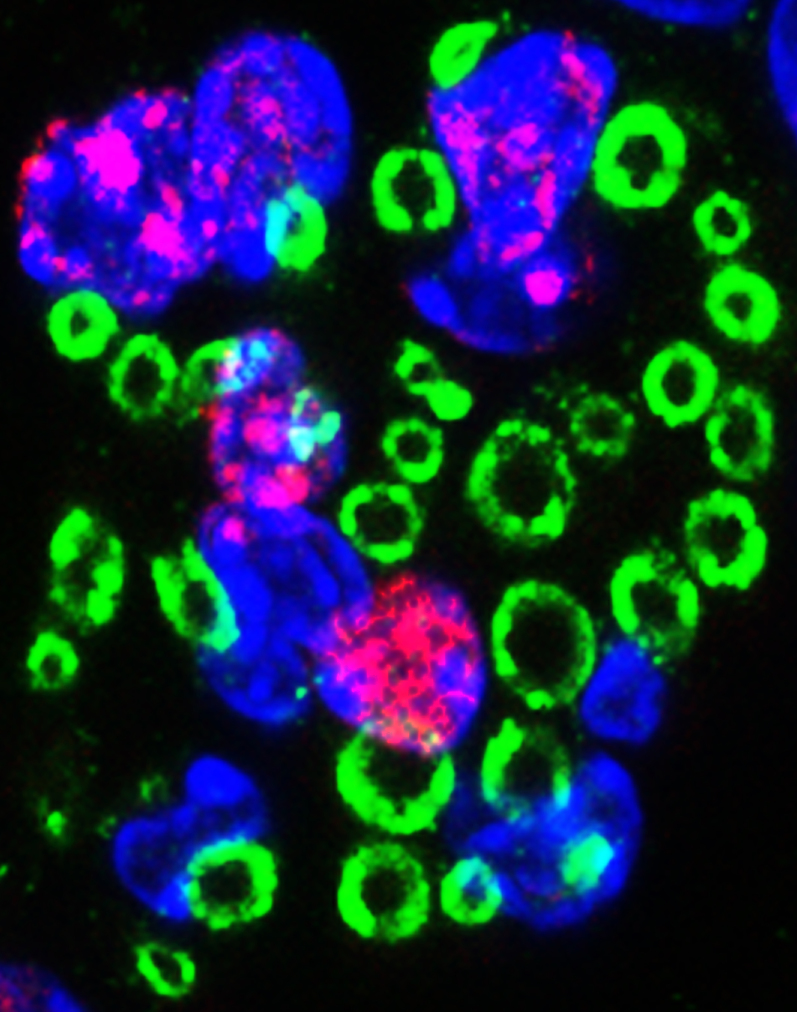
Spontaneous remyelination after spinal cord injury is mediated largely by Schwann cells of unknown origin. Bartus *et al*. show that neuregulin-1 promotes differentiation of spinal cord-resident precursor cells into PNS-like Schwann cells, which remyelinate central axons and promote functional recovery. Targeting the neuregulin-1 system could enhance endogenous regenerative processes.

## Introduction

Spinal cord injury comprises many pathological events and leads to devastating deficits in bodily functions. Apart from loss of motor function and paralysis, many patients also suffer incontinence (Potter, 2006), chronic pain (Ravenscroft *et al.*, 2000) and psychiatric disorders (Chevalier *et al.*, 2009). The financial burden of spinal cord injury is staggering, with healthcare costs among the highest of any medical condition (Cannon, 2013) and improving neurological outcome after spinal cord injury remains a major clinical challenge (Dietz and Fouad, 2014; Ramer *et al.*, 2014).

The complex pathophysiology of spinal cord injury is biphasic, comprising primary trauma followed by secondary injury progression. Initial tissue disruption, haemorrhaging and oxidative stress are followed by inflammation, death of neurons and glia, axonal demyelination and degeneration, extracellular matrix remodelling, glial scar formation, and cavitation (Schwab and Bartholdi, 1996; Hagg and Oudega, 2006; Fitch and Silver, 2008; Gaudet and Popovich, 2014). Targeting any of these injury-induced reactive changes, singularly or in combination, may contribute to improved neurological outcome after spinal cord injury and lead to new therapeutic strategies (Oudega *et al.*, 2012; Ramer *et al.*, 2014). Despite the severe neurological deficits, some degree of spontaneous, but incomplete, functional recovery is observed in almost all cases (Fawcett *et al.*, 2007). In terms of underlying biology, it is apparent that a number of spontaneous regenerative events occur after spinal cord injury, including neurogenesis, plasticity and remyelination of spinal axons (Beattie *et al.*, 1997; Raineteau and Schwab, 2001; Weidner and Tuszynski, 2002; Hagg and Oudega, 2006). It is crucial to better understand the cellular and molecular mechanisms underlying these spontaneous repair events, which might provide a route to enhance them directly and to design combinations of effective therapeutic interventions.

Sparing of some subpial axon-containing spinal tissue around the lesion core is a typical feature of traumatic spinal cord injury (Crowe *et al.*, 1997; Norenberg *et al.*, 2004; Guest *et al.*, 2005). In the spared tissue, viable axons are observed but these are unable to conduct under normal physiological conditions (Koles and Rasminsky, 1972; Nashmi and Fehlings, 2001; James *et al.*, 2011). This axonal impairment is associated with acute and profound demyelination of spinal axons after traumatic contusion-type spinal cord injuries (Bunge *et al.*, 1960; James *et al.*, 2011; Plemel *et al.*, 2014; Papastefanaki and Matsas, 2015), which are the most common form of spinal cord injury suffered by humans (Norenberg *et al.*, 2004). Apoptotic loss of oligodendrocytes and gradual myelin degradation are among the major events that follow primary damage in rodents and humans (Crowe *et al.*, 1997; Beattie *et al.*, 2002; Buss *et al.*, 2005). Remyelination of demyelinated axons within the injury penumbra is an important regenerative process described in several animal models of spinal cord injury (Bunge *et al.*, 1961; McDonald and Belegu, 2006; James *et al.*, 2011; Plemel *et al.*, 2014; Papastefanaki and Matsas, 2015). However, this endogenous repair is suboptimal and incomplete. Chronically demyelinated axons have been reported in animal models (Blight and Decrescito, 1986; Waxman, 1989; James *et al.*, 2011) and are observed up to a decade after human spinal cord injury (Bunge *et al.*, 1993; Guest *et al.*, 2005). Myelin integrity is essential for CNS physiology, allowing fine tuning of motor skills and sensory integration, and there is evidence that remyelination restores efficient signal conduction and functional outcome (Smith *et al.*, 1979; Duncan *et al.*, 2009) and is crucial for axonal protection and metabolic support (Franklin and Ffrench-Constant, 2008; Irvine and Blakemore, 2008). Strategies to prevent demyelination at early stages and to accelerate and enhance myelin repair at later stages are an important part of the wide spectrum of promising therapies for spinal cord injury (Plemel *et al.*, 2014; Ramer *et al.*, 2014; Papastefanaki and Matsas, 2015).

An interesting phenomenon in the injured CNS is remyelination of central axons by Schwann cells, which are normally excluded from the CNS and represent the primary myelinating cells in the peripheral nervous system (PNS). This unexplained intrinsic myelin repair is also observed in the chronically injured human spinal cord, where demyelinated axons become associated with peripheral myelin, most noticeably in the dorsal columns (Bunge *et al.*, 1993; Guest *et al.*, 2005). Evidence demonstrates that Schwann cells that myelinate CNS axons produce functional and stable myelin that has normal properties (Blight and Young, 1989; Felts and Smith, 1992, 1996). In addition, in the remyelinated axons the composition of the nodes of Ranvier is normal (Black *et al.*, 2006). Two distinct mechanisms might account for the presence of ‘central’ Schwann cells: peripheral Schwann cells might enter the spinal cord as a result of injury to the transition zone between PNS/CNS and/or they may be derived from CNS-resident oligodendrocyte precursors by (trans)-differentiation (Sims *et al.*, 1998; Jasmin *et al.*, 2000; Zawadzka *et al.*, 2010; Perlin *et al.*, 2011). Independent of the cellular mechanisms, molecular mechanisms that govern the Schwann cell-mediated remyelination of injured spinal axons are unknown. Understanding the molecular events involved in this process is important and could be exploited to enhance function of surviving axons, or improve function of regenerating axons, in the injured spinal cord and thus improve functional recovery. Such mechanisms might also promote repair in other CNS disorders that involve a demyelinating pathology.

The growth factor neuregulin 1 (Nrg1, encoded by *NRG1*), which signals via ErbB tyrosine kinase receptors, is crucial for Schwann cell development and function in the PNS. Nrg1 stimulates survival of precursors, migration of Schwann cells, ensheathment, myelination and remyelination of peripheral axons (Birchmeier and Nave, 2008; Fricker and Bennett, 2011; Stassart *et al.*, 2013; Nave and Werner, 2014). There are over 30 different Nrg1 isoforms, all of which possess an epidermal growth factor (EGF)-like signalling domain essential for receptor binding and biological activity. Isoforms differ in their functional role and expression and can be classified according to the structure of their N termini (Falls, 2003; Newbern and Birchmeier, 2010). Isoforms containing an immunoglobulin (Ig)-like domain (types I and II) can be either directly secreted or released as soluble proteins from the cell surface after proteolytic cleavage. Isoforms possessing a cysteine-rich domain (type III isoforms) have two transmembrane domains and require proteolytic cleavage by BACE1 (β-site APP-cleaving enzyme 1) for full activity. *In vivo* these isoforms are thought to be retained on the cell membrane and signal in a juxtacrine manner (Hu *et al.*, 2006; Willem *et al.*, 2006) although type III isoforms can undergo dual cleavage by BACE1 and ADAM17 to release the EGF domain, which signals in a paracrine fashion (Fleck *et al.*, 2013). We hypothesized that Nrg1 may be a key regulatory factor for spontaneous remyelination of injured axons in the CNS by Schwann cells. Nrg1 is also known to modulate oligodendrocyte function (Brinkmann *et al.*, 2008; Taveggia *et al.*, 2008; Makinodan *et al.*, 2012; Lundgaard *et al.*, 2013) and neural precursor cells in the spinal cord have been shown to be responsive to Nrg1, as exogenous Nrg1 promotes oligodendrocyte production and repair (Zhang *et al.*, 2011; Gauthier *et al.*, 2013). To determine the mechanisms controlling remyelination of CNS axons by Schwann cells after spinal cord injury and to avoid confounds of the developmental functions, we introduced Nrg1 mutation in adult mice using a tamoxifen inducible Cre (Meyer and Birchmeier, 1995; Fricker *et al.*, 2013). To determine the importance of Nrg1 in repair, we used a clinically relevant spinal cord contusion injury model (James *et al.*, 2011), and compared myelination in the injured spinal cord and functional outcome in the presence and absence of Nrg1. To determine the importance of specific Nrg1 isoforms in Schwann cell-mediated remyelination after traumatic spinal cord injury, we also examined this regenerative process after ablating Ig-containing isoforms of Nrg1 (IgNrg1) only, which leaves the type III Nrg1 intact.

We demonstrate that Nrg1 is essential for remyelination of dorsal column axons by PNS-like Schwann cells and that an absence of Nrg1 elicits a profound and persistent demyelinating phenotype and axonal conduction failure after spinal cord injury. IgNrg1 isoforms are dispensable for this process, implicating type III Nrg1 as the key isoform mediating this process. We also provide evidence that the majority of centrally remyelinating Schwann cells derive from newly generated precursor cells within the spinal cord. Interference with Nrg1 signalling also significantly impacts the degree of spontaneous locomotor recovery after contusive spinal cord injury. These data reveal that Nrg1 signalling mediates an endogenous regenerative event in which Schwann cells remyelinate denuded central axons after traumatic spinal cord injury and that Nrg1 is an important mediator of spontaneous functional repair after spinal cord injury.

## Materials and methods

### Animals

All animal work carried out conformed to UK Home Office legislation (Scientific Procedures Act 1986). CAG-Cre-ER^TM^; Nrg1^fl/fl^ mice, and CAG-Cre-ER^TM^; IgNrg1^fl/fl^ were bred by crossing Nrg1^fl/fl^ mice and IgNrg1^fl/fl^ with CAG-Cre-ER^TM^ mice [JAX(r) mice 004682], respectively; as described previously (Cheret *et al.*, 2013; Fricker *et al.*, 2013). The generation and genotyping of mutant mice with floxed alleles of *Nrg1* (Nrg1^fl/fl^) mice has previously been described (Yang *et al.*, 2001; Brinkmann *et al.*, 2008; Fricker *et al.*, 2013). These mice are null for α-isoforms of *Nrg1* in the absence of Cre recombination as they carry a premature stop codon in exon 7, which encodes the α-EGF domain (Li *et al.*, 2002). The loxP sites flank exons 7–9, and exon 8 encodes the β-EGF domain. Cre recombination therefore results in ablation of all remaining β isoforms. CAG-Cre-ER^TM^ construct detection and expression evaluation is described previously (Fricker *et al.*, 2013). The generation and genotyping of IgNrg1^fl/fl^ has been described previously (Cheret *et al.*, 2013); briefly, mutant alleles have the exon 4 flanked by loxP sites, exons 3 and 4 of *Nrg1* encode the Ig-like domain, mutations specifically interfere with the production of IgNrg1 forms (i.e. Nrg1 type I and Nrg1 type II), but not CRD-Nrg1 transcripts. In CAG-Cre-ER^TM^ mice, a tamoxifen inducible form of Cre recombinase is expressed ubiquitously driven by a chimeric promoter constructed from a cytomegalovirus intermediate-early enhancer and a chicken β actin promoter/enhancer (Hayashi and McMahon, 2002). Conditional Nrg1 (conNrg1 mutant) and conIgNrg1 mice were generated by administering tamoxifen (Sigma T5648, 0.25 mg/g body weight in corn oil) by oral gavage for five consecutive days to 10-week-old CAG-Cre-ER^TM^; Nrg1^fl/fl^ and CAG-Cre-ER^TM^; IgNrg1^fl/fl^ mice respectively. Tamoxifen was administered 4 weeks prior to surgery. We have previously assessed Nrg1 expression in conNrg1 mutant within spinal cord at 4 weeks following this treatment regimen: conNrg1 demonstrate a 83% reduction in the expression of the βEGF domain of Nrg1 (which is critical for biological activity of all isoforms) relative to control (Fricker *et al.*, 2013) and on assessment of the most abundant Nrg1 type III isoform protein there was a significant reduction in both the full length and the cleaved C-terminal fragment. For experiments with CAG-Cre-ER^TM^; Nrg1^fl/fl^ two types of control animals were used for comparison: vehicle controls, which were CAG-Cre-ER^TM^; Nrg1^fl/fl^ littermates treated with corn oil alone or tamoxifen control, which were tamoxifen treated Nrg1^fl/fl^ littermates. For experiments with CAG-Cre-ER^TM^; IgNrg1^fl/fl^ mice we used tamoxifen control littermates. Wherever possible, we included equal numbers of animals of each gender in each experimental group.

### Spinal contusion injury

#### Mice

Mice were anaesthetized with isoflurane, their backs were shaved and cleansed, and core temperature was maintained close to 37 °C using a self-regulating heated blanket. Single doses of 0.05 mg/kg buprenorphine and 5 mg/kg carprofen were administered subcutaneously at the time of induction and the morning after surgery. Animals underwent midthoracic laminectomy and received a moderate midline 50 kdyne spinal contusion injury through the intact dura at spinal level T10/11 using an Infinite Horizon’s impactor (Precision Systems Instrumentation). Overlying muscle and skin were sutured in layers, subcutaneous saline was administered, and animals were left to recover from anaesthesia in a 37 °C incubator. Saline and enrofloxacin (5 mg/kg) were given subcutaneously daily for 3 and 7 days, respectively, after injury. Bladders were manually expressed three to four times daily during the first 2 to 3 days after surgery and twice daily thereafter until the end of the study period.

#### Rats

Adult female Sprague Dawley rats (150–200 g; Harlan Laboratories) were used, housed under a 12-h light/dark cycle with *ad libitum* access to food and water. Animals were anaesthetized using a mixture of ketamine (60 mg/kg) and medetomidine 0.25 mg/kg, administered intraperitoneally. Following midthoracic laminectomy to expose the spinal cord leaving the dura intact, animals received a moderate midline 150 kdyne spinal contusion injury at spinal level T10/11 using an Infinite Horizon’s impactor (Precision Systems Instrumentation) (James *et al.*, 2011). Postoperative care was performed as above with the exception that bladders were manually expressed twice daily until reflexive emptying returned (typically 6 to 9 days after injury).

### Spinal contusion injury with dorsal root removal

Adult female Sprague Dawley rats (150–200 g; Harlan Laboratories) were used and contusion surgeries and postoperative care were performed as described above. One cohort of animals received moderate 150 kdyne T10/T11 contusions (*n = *6) only. A second cohort of animals received moderate 150 kdyne T10/T11 contusions followed by bilateral dorsal root removal at thoracic levels T9 to T11/T12 (*n = *8). To ascertain complete removal, roots were post-fixed overnight in 4% paraformaldehyde in 0.1 M phosphate buffer and whole mounts were prepared for immunostaining for glial fibrillary acidic protein (GFAP) to label reactive astrocytes in the region of the dorsal root entry zone (rabbit polyclonal anti-GFAP, 1:2000, DakoCytomation). This experiment was carried out in rats due to the complexity of the surgery and low survival rate of mice (mainly caused by blood loss following lateral bone removal that is necessary to sufficiently expose roots). However, electron microscopy data and immunohistochemistry confirmed that the pattern and time course of Schwann cell-mediated remyelination is the same in mice and rats (James *et al.*, 2011; and see Figs 2 and 5).

### Behavioural assessments

#### Basso Mouse Scale

The Basso Mouse Scale (BMS) (Basso *et al.*, 2006) was used to assess open field hindlimb locomotor function (*n = *7–9 per group). This involved placing the animal in a circular open field (diameter ∼1 m) and assessing both hindlimbs during locomotion (over a 4-min session). Scores were calculated according to the 10 point (0–9) BMS scale. For further detailed assessments of differences between conNrg1 and conIgNrg1 null mice in additional cohorts of animals, mice were assessed with the 12 point (0–11) BMS subscore scale, which further delineates recovery of specific locomotor features that may not be apparent in the overall BMS score (such as quantifying improvements in the areas of stepping frequency, coordination, paw position, trunk stability, and tail position). Testing was performed by two experimenters blinded to the treatment groups on Days 2, 5 and 7 after injury and once weekly thereafter for 8 weeks, followed by sacrificing animals for immunohistochemical and ultrastructural analysis. All BMS behavioural data are presented as mean ± standard error of the mean (SEM) values and statistical significance was accepted with *P < *0.05 using two-way ANOVA with Bonferroni *post hoc* tests (BMS score) or one-way ANOVA with Tukey’s *post hoc* tests (BMS subscore).

#### Inclined beam-walking test

For further detailed assessments of differences between conNrg1 and conIgNrg1 null mice, animals were also assessed on the inclined beam walking test. Beam-walking apparatus consisted of an inclined beam (100 cm) fixed to a black ‘goal box’. The horizontal inclined beam consisted of a flat surface that gradually narrowed (1.5 cm at the widest; 0.5 cm at the narrowest) and a small ledge underneath on either side. Animals were trained for seven consecutive days before baseline readings were obtained. Left and right hind limb scores were calculated based on number of weight supported steps taken on the beam as well as lower scores for steps taken on the small ledges. The beam was divided into quarters; one point was scored for a weight supported step on the beam in the first broadest division. This score was doubled, tripled or quadrupled in the second, third and fourth sections of the beam due to the increased difficulty of the tapered beam. In all sections one point was scored for a step taken on the small ledges. Data (*n = *6–9 per group) are presented as mean ± SEM values and statistical significance was accepted with *P < *0.05 using one-way ANOVA with Tukey’s *post hoc* tests.

### Electrophysiology

To carry out electrophysiological assessment of dorsal column function, mice were deeply anaesthetized with urethane [0.1 ml/10 g of 12.5% solution administered intraperitoneally (i.p.)] and depth of anaesthesia was regularly assessed by monitoring withdrawal reflexes and respiratory rate. Core temperature was maintained close to 37 °C using a self-regulating heating pad. A laminectomy was performed to expose spinal tissue from 5 mm rostral to 5 mm caudal of the contusion injury site. The sural nerve of the left hindlimb was exposed and freed from connective tissue, and all nervous tissue was covered with mineral oil. Silver ball stimulating electrodes were then placed at 5 mm rostral and 5 mm caudal of the injury site and a pair of silver wire recording electrodes was hooked underneath the sural nerve with a separation of ∼3 mm between the wires. To assess the extent of sensory conduction through the spinal contusion injury site, recordings were made from the sural nerve (an almost exclusively sensory nerve) while first stimulating the spinal cord caudal to the lesion and then repeating this procedure whilst stimulating rostral to the lesion site. Stimulation was delivered in 250 µs square wave pulses at a frequency of 0.5 Hz and at a supramaximal intensity (typically 600–800 µA). Amplitude analysis was carried out on traces averaged from 16 recordings taken following stimulation at each site (rostral or caudal to lesion). The peak-to-peak amplitude of the averaged potential recorded whilst stimulating rostral to the lesion site was then calculated as a percentage of the averaged potential recorded whilst stimulating caudal to the lesion, thus estimating the percentage of sensory fibres in the sural nerve projecting beyond the injury site that remain capable of conduction. Although the stimulation technique used here is likely to have activated more than just the fibres of the dorsal columns, the sural nerve is almost exclusively composed of sensory fibres and therefore only the activity of long distance afferent fibres will be recorded using this protocol. All recordings were made using a PowerLab unit (AD Instruments) and amplitude analysis was carried out using LabChart 8 software (AD Instruments). Data (*n = *5–6 per group) are presented as mean ± SEM values and statistical significance was accepted with *P < *0.05 using one-way ANOVA with Tukey’s *post hoc* test.

### Tissue preparation and immunohistochemistry

Animals were deeply anaesthetized with sodium pentobarbital (Euthatal: 80 mg/kg, i.p) and transcardially perfused with phosphate-buffered saline (PBS) (containing heparin) followed by 4% paraformaldehyde in 0.1 M phosphate buffer containing 1.5% picric acid. Immediately after perfusion, lesion site tissue was dissected (∼10 mm with the lesion epicentre located centrally). Tissue was post-fixed overnight at 4 °C, cryoprotected in 20% sucrose for 48–72 h, then embedded and frozen in O.C.T. before being cut into serial transverse (20 µm) sections. Sections were immunostained using the following primary antibodies: rabbit polyclonal anti-glial fibrillary acidic protein (GFAP) to label reactive astrocytes (1:2000, DakoCytomation), chicken polyclonal anti-protein zero (P0) to label Schwann cell-associated myelin (1:500, Abcam), chicken polyclonal anti-proteolipid protein (PLP) to label oligodendrocyte-associated myelin (1:200, Millipore), rabbit polyclonal anti-neurofilament 200 (NF200) to label axons (1:200, Sigma), rabbit polyclonal anti-laminin to visualize Schwann cell basal lamina (1:1000, Dako), and rabbit polyclonal anti-Olig2, a marker for oligodendrocytes (1:500, Millipore). Complementary secondary antibodies were goat anti-chicken biotin (1:400, Abcam), ExtrAvidin FITC conjugate (1:500, Sigma), goat anti-chicken Alexa 488 (1:1000, Invitrogen), goat anti-rabbit Alexa 568 (1:1000, Invitrogen) and goat anti-rabbit Alexa 488 (1:1000, Invitrogen). Briefly, after blocking with 10% goat serum in PBS containing 0.2% Triton™ X-100 (PBST) for 1 h at room temperature, the sections were incubated in PBST containing primary antibodies overnight at room temperature. After four washes of 5 min with PBS, sections were incubated in PBST containing complementary secondary antibodies for 4 h at room temperature. After four washes of 5 min in PBS, sections were coverslipped with Vectashield mounting medium (Vector Laboratories). Images were acquired using Nikon A1R Si Confocal Imaging system on an Eclipse Ti-E inverted microscope.

For haematoxylin and eosin staining, spinal sections were rinsed in tap water, stained with haemalum for 5 min and then rinsed in running tap water until clear. Slides were then dipped five times into 0.5% hydrochloric acid in 70% IMS (acid-alcohol) and quickly returned to running tap water for 1 min, placed in eosin for 5 min, returned to tap water, dehydrated and mounted using DPX. Using this technique, nuclei and any basophilic components are labelled blue, while components such as cytoplasm and collagen are labelled as shades of pink-orange.

### Labelling of cellular DNA with EdU and EdU staining

Wild-type mice (*n = *3) received spinal contusion injuries, as above, followed by administration of 5-ethynyl-2’-deoxyuridine (EdU, Invitrogen) (Salic and Mitchison, 2008; Zeng *et al.*, 2010), administered by intraperitoneal injection (200 µg per injection) for 10 consecutive days beginning at 24 h post-injury. Control uninjured mice (*n = *3) received the equivalent EdU dosing paradigm. At 4 weeks post-injury mice were deeply anaesthetized with sodium pentobarbital (Euthatal: 80 mg/kg, i.p) and transcardially perfused with PBS (containing heparin) followed by 4% paraformaldehyde in 0.1 M phosphate buffer, and spinal cords were harvested and prepared for immunohistochemistry. Tissue was post-fixed with 4% paraformaldehyde in 0.1 M PBS for 3 h at 4 °C, cryoprotected for 24 h at 4 °C in 30% sucrose in PBS prior to embedding and sectioned at 30-µm thickness. EdU staining was conducted using the Click-iT^TM^ EdU imaging kit (Invitrogen) according to the manufacturer’s protocol but adapted for immunohistochemical double-staining of spinal cord tissue. Slides containing mounted frozen spinal cord sections were allowed to thaw, and then rehydrated with PBS. After rehydration with PBS the sections were incubated in 10% normal donkey serum permeabilization/blocking buffer made in PBS containing 0.3% Triton™ X-100 for 15 min. This was followed by one PBS rinse and incubation with EdU detection solution (Invitrogen) for 1 h at room temperature. Slides were then washed three times with PBS before incubation with permeabilization/blocking buffer overnight at room temperature. Subsequently, primary antibodies made in PBST to label either P0 or PLP were incubated overnight for ∼24 h at room temperature. After four washes of 5 min with PBS, sections were incubated in PBST containing complementary secondary antibodies for 4 h at room temperature. After four washes of 5 min in PBS, sections were coverslipped with Vectashield mounting medium (Vector Laboratories). Images were acquired using Nikon A1R Si Confocal Imaging system on an Eclipse Ti-E inverted microscope.

### Immunohistochemistry image analysis

Quantification of P0 and PLP in mouse spinal cords (*n = *4–5 per group) was carried out by measuring the immunopositive areas in the dorsal column (AxioVision LE software), which were then expressed as % of total dorsal column area. Total dorsal column area was measured by taking the mean area of intact dorsal column, measured rostral and caudal to the lesion site. Quantification of P0 in the lesion epicentre in rat spinal cords with or without multiple dorsal root removal (*n = *4–5 per group) was carried out by measuring the immunopositive P0 cluster in the dorsal column, which was then expressed as per cent of total dorsal column area as above. Quantification of Olig2 (*n = *3 per group) was carried out by unbiased particle counts following background subtraction (ImageJ software). Images were acquired sequentially, using the same exposure parameters. All anatomical quantification was carried out by an experimenter blinded to the treatment group and data are expressed as mean ± SEM values, using one-way ANOVA with Tukey’s *post hoc* tests.

### Electron microscopy

Animals were terminally anaesthetized using sodium pentobarbital (Euthatal; 80 mg/kg, i.p.) and transcardially perfused with 0.9% saline followed by 3% glutaraldehyde and 4% paraformaldehyde in 0.1 M phosphate buffer, a section of thoracic spinal cord was removed (∼10 mm) with the lesion epicentre located centrally. Two to three millimetre sections were taken from the lesion epicentre and from the rostral and caudal lesion borders and postfixed in the same fixative buffer (3% glutaraldehyde and 4% paraformaldehyde in 0.1 M phosphate buffer) for a minimum of 48 h at 4 °C and processed as previously described (James *et al.*, 2011; Fricker *et al.*, 2013). Semithin and ultrathin sections were cut and stained by the Centre for Ultrastructural Imaging (King’s College London, London, UK). Ultrathin sections were mounted on unsupported gilded copper grids (150-square mesh) from TAAB and were visualized on a Hitachi H7600 transmission electron microscope. For analysis, photomicrographs of the region containing the ascending dorsal column projection from each animal were taken at ×8000 magnification, the area of which totalled at least 50% of the total area of the cross-section of the dorsal column. Full montages of grid squares were taken (∼100 pictures per mesh) and randomly chosen images from a given grid square were analysed. The total number of axons, total number of myelinated axons, Schwann cell-myelinated axons, and oligodendrocyte-myelinated axons (using the criteria described in Supplementary Fig. 5) were counted from these montages of grid squares and normalized to the dorsal column area. To calculate G-ratios, axon diameters and non-myelinated axons with a diameter >1 µm from ∼30 individual pictures at ×8000 magnification were randomly chosen per animal; analysis was performed on all of the axons within each picture and axon diameter and G-ratio (axon diameter/fibre diameter) were calculated using AxioVision LE Rel. 4.2 Software. At least 750 axons were measured per animal. The examiner was blind to the genotype. The one-way ANOVA using the Tukey *post hoc* test was used for comparison of more than two groups. Cumulative frequencies were compared statistically using the Kolmogorov-Smirnov test.

### Fluorescence *in situ* hybridization and immunohistochemistry

Uninjured (*n = *3) and injured (4 weeks post-injury; *n = *3) wild-type mice were deeply anaesthetized with sodium pentobarbital (Euthatal: 80 mg/kg, i.p) and transcardially perfused with DPEC-treated sterile PBS followed by 4% paraformaldehyde in 0.1 M phosphate buffer, and spinal cords were harvested and prepared for immunohistochemistry. Tissue was post-fixed with 4% paraformaldehyde in 0.1 M PBS for 3 h at 4 °C, cryoprotected for 24 h at 4 °C in 30% sucrose in diethyl pyrocarbonate-treated PBS prior to embedding and sectioned at 30-µm thickness. Pan-Nrg1 *in situ* probes (Meyer *et al.*, 1997) were transcribed *in vitro* and labelled with digoxygenin (DIG) according to manufacturer’s instructions (Roche). Following overnight hybridization at 65 °C, sections were incubated with anti-DIG antibodies (Roche) and developed as previously described (Hopman *et al.*, 1998). To examine Nrg1 expression in different cell types, slides were then co-stained for P0, Olig2 and NeuN immunohistochemically, as described above.

### Extraction of fresh tissue

Spinal cord (lesion epicentre) and L4 and 5 DRGs were extracted from animals at 1 and 4 weeks after injury, snap frozen on liquid nitrogen and stored at −80 °C. The same tissue from naïve animals was used as control. Tissue samples were then homogenized and total RNA obtained using a ‘hybrid’ method of phenol extraction (TriPure, Roche) and column purification (High Pure RNA tissue Kit, Roche) according to manufacturer’s protocols. All samples were DNase treated to prevent genomic contamination. RNA was subsequently synthesized into cDNA using Transcriptor Reverse Transcriptase (Roche) following the manufacturer’s protocol.

### Quantitative polymerase chain reaction

Quantitative PCR (qPCR) was performed using the LC 480 system. All primers used, shown in Supplementary Table 1, had an efficiency of 100 ± 10% and were designed using primer blast unless stated otherwise. Gene expression levels were measured using the ΔΔCT method and normalized against the reference genes *Gadph* and *Hprt1*. The relative mRNA expression is shown as the amount of transcript in injured samples versus naive controls. The one-way ANOVA using the Tukey’s *post hoc* test was used for comparison of more than two groups. When the values are not normally distributed the one-way ANOVA on ranks *post hoc* Dunn’s method was used.

## Results

### Ablation of Nrg1 prevents spontaneous remyelination of axons in the injured spinal cord

**Figure 1:**
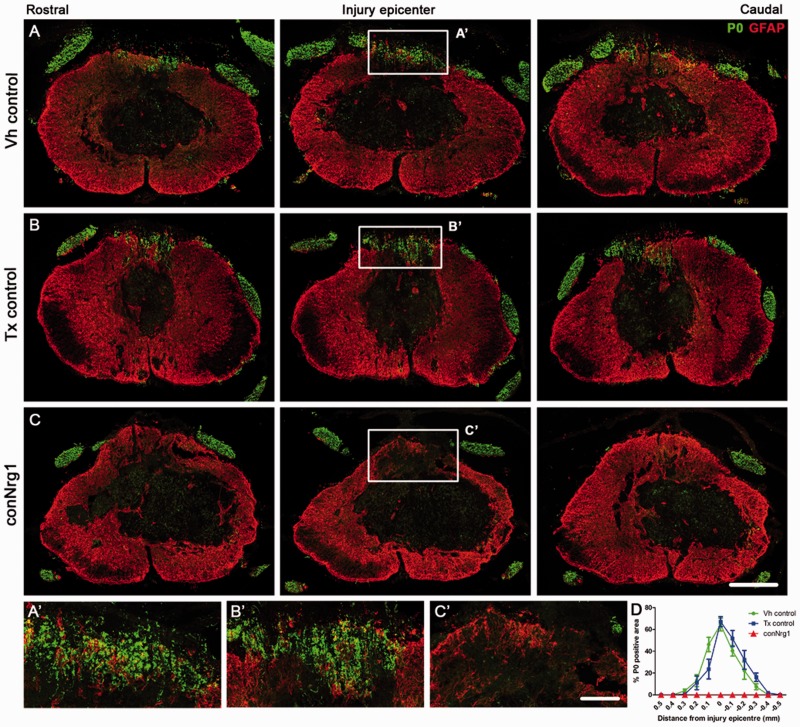
**Ablation of Nrg1 prevents remyelination of spinal axons by Schwann cells after spinal cord injury.** (**A–C**) Co-staining of astrocytes (GFAP, red) and Schwann cell-associated myelin (P0, green) in serial sections of the spinal cord that span the rostrocaudal axis of the injury in vehicle control (Vh control, **A**), tamoxifen control (Tx control, **B**) and Nrg1-ablated (conNrg1, **C**) contused mouse spinal cords at 10 weeks post-injury. In all animals, the peripheral myelin protein P0 is apparent outside the spinal cord, in the peripheral dorsal and ventral roots, as expected. However, 10 weeks after contusion injury, Schwann cell-associated myelin (P0) is also observed in the spinal dorsal columns of control animals, being particularly abundant in the epicentre of the lesion (**A** and **B**). Strikingly, P0 is absent in the spinal dorsal columns of injured mice lacking Nrg1 (conNrg1; **C**). (**A’**–**C’**) High magnification of boxed areas indicated in **A**–**C**. (**D**) Quantification of P0-positive area in the dorsal columns assessed in sections that span the rostrocaudal axis of the injury site reveals undetectable levels of P0 in conNrg1 mice, compared to control groups. Data are presented as mean ± SEM. (**P < 0.007, two-way ANOVA, *post hoc* Bonferroni *n = *4–5 animals/group). Scale bars = 250 µm (**C**); 50 µm (**C’**). Images not using the red/green colour scheme are available in the Supplementary material.

The molecular control of Schwann cell-mediated axonal remyelination in the injured CNS is unknown. We asked whether Nrg1 is required for Schwann cell-mediated remyelination of dorsal column axons after spinal contusion injury, and whether Nrg1 ablation influences the degree and nature of remyelination. We first confirmed that there were no differences in the expression of global astrocyte and axonal markers (GFAP and NF200, respectively) or in peripheral and central myelin expression (P0 and PLP, respectively) in uninjured control mice and mice with conditional Nrg1 mutations (hereafter called conNrg1 mice) (Supplementary Fig. 1). Immunoreactivity for P0 identifies Schwann cell-derived peripheral myelin and is normally only expressed in the PNS. As expected, P0 was detected in the peripheral dorsal and ventral roots but was absent from the spinal cord of all uninjured animals (Supplementary Fig. 1A and C). However, 10 weeks after contusive spinal cord injury, P0 was present in the dorsal columns of the injured spinal cord in both vehicle- and tamoxifen-treated control animals. P0 was most abundant at the lesion epicentre, where ∼60% of the dorsal column area expressed Schwann cell-associated myelin (Fig. 1A, B, D and Supplementary Fig. 2). Strikingly, there was no P0 immunoreactivity within the injured spinal cord of conNrg1 mice. Thus, when all Nrg1 isoforms are ablated, Schwann cell-associated myelin in the spinal dorsal columns is completely absent after injury (Fig. 1C, D and Supplementary Fig. 2). Interestingly, the presence of Schwann cell myelin within the spinal cord after injury was only observed in the dorsal column region of the spinal cord and not in other white matter tracts or in the cellular and matrix filled lesion core (Supplementary Figs 2 and 3).

To determine whether abrogation of Schwann cell-mediated remyelination may trigger compensatory remyelination by oligodendrocytes, we examined the expression of PLP, the major protein of oligodendrocyte-derived myelin which is expressed exclusively in the CNS and absent from peripheral tissues (Supplementary Fig. 1B and D). PLP immunohistochemistry revealed that the absence of Schwann cell-mediated remyelination in conNrg1 mutants did not evoke compensatory myelination by oligodendrocytes, with a similar pattern of PLP expression observed at 10 weeks after spinal cord injury in conNrg1, vehicle- or tamoxifen-treated control animals (Supplementary Fig. 4). In particular, abundant PLP staining was observed in white matter tracts of the spinal cord throughout the rostrocaudal axis which was dramatically reduced in the dorsal columns at the injury epicentre (Supplementary Fig. 4D). Double labelling revealed that areas normally associated with abundant P0 immunoreactivity in control injured animals (Fig. 1A’ and B’) were positive for the axonal marker NF200 but devoid of central myelin (Supplementary Fig. 4A’ and B’), while in conNrg1 injured animals these areas remained negative for both Schwann cell-derived (P0, Fig. 1C’) and oligodendrocyte-derived (PLP, Supplementary Fig. 4C’) myelin. To confirm that there was no change in the number of oligodendrocytes, we also assessed the expression of the transcription factor Olig2, which is an essential regulator of oligodendrocyte development. Consistent with unchanged distribution of PLP, no differences in Olig2 expression were observed between control and conNrg1 mice at 4 weeks after injury (Supplementary Fig. 5A–C). At this stage, remyelination by PNS-like Schwann cells is normally evident (James *et al.*, 2011; Fig. 5C and F). The profound interference with Schwann cell-mediated remyelination of dorsal column axons suggests that Nrg1 is a key regulator of spontaneous myelin repair.

### Ultrastructural analysis reveals profound demyelination in the injured spinal cord after Nrg1 ablation

**Figure 2:**
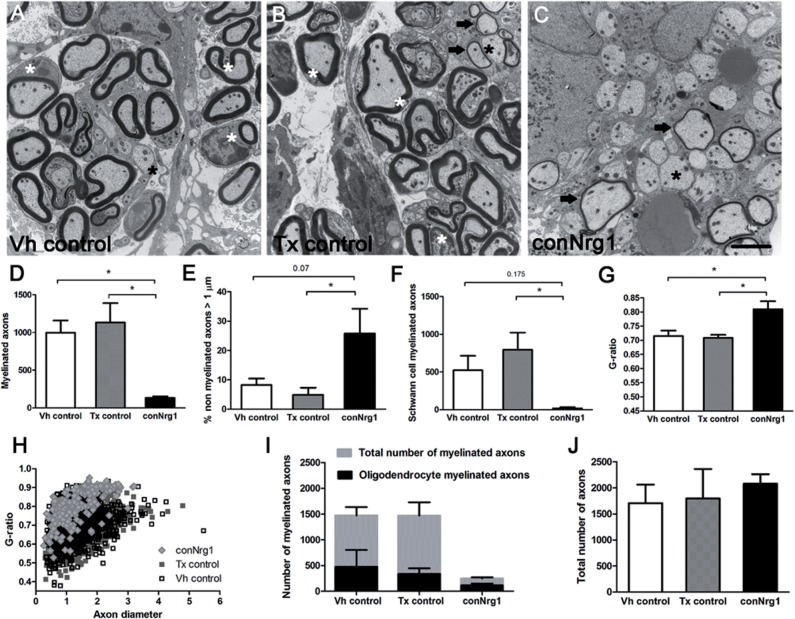
**Ultrastructural analysis of Schwann cell-mediated remyelination of spinal axons after injury and its dependence on Nrg1 signalling.** (**A**–**C**) Electron micrographs of transverse sections of the dorsal column 10 weeks after spinal contusion injury in vehicle control (Vh control; **A**), tamoxifen control (Tx control; **B**) and conditional Nrg1 mutant (conNrg1; **C**) mice. In control animals, axons are undergoing remyelination and Schwann cells (white asterisk) can be seen to mediate remyelination; Schwann cells and their myelin are identified by the signet ring-like appearance of Schwann cell myelin, thicker and more compact myelin, and basal laminae around the Schwann cells. In conNrg1 mutant animals, many large diameter unmyelinated axons are visible (black asterisk), Schwann cells were rarely detected and the small degree of remyelination is mediated by oligodendrocytes that produce a thin myelin sheath (arrows). Remyelinating oligodendrocytes do not have nuclei directly apposed to the myelin or surrounding basal lamina and oligodendrocyte-associated myelin is less dense than the myelin associated with Schwann cells. (**D**) The number of myelinated axons in the dorsal column was significantly decreased in conNrg1 mice compared with control (Vh control = 998 ± 160, Tx control = 1132 ± 256 and conNrg1 = 134 ± 20). (**E**) The percentage of unmyelinated axons with a diameter >1 µm was increased in conNrg1 animals versus control (Vh control = 8% ± 2, Tx control = 5% ± 2 and conNrg1 = 26% ± 8). (**F**) A dramatic reduction in the number of Scwann cell-myelinated axons in conNrg1 mutant animals was observed (Vh control = 522 ± 192, Tx control = 794 ± 227 and conNrg1 = 17 ± 16). (**G**) A significant increase in the G-ratio in the conNrg1 animals (Vh control = 0.72 ± 0.01, Tx control = 0.71 ± 0.02 and conNrg1 = 0.81 ± 0.02) indicate very thin myelin sheaths. Data shown in **D**–**G** are presented as mean ± SEM. (*P < 0.05, one-way ANOVA, *post hoc* Tukey’s *n = *3–4 animals/group). No significant differences were observed between the two control groups in any of the measures analysed. Scale bar = 2 µm. (**H**) Scatter plot relating G-ratio and axon diameter of all the axons analysed show a shift to higher G-ratios in conNrg1 animals (*P < *0.001 Kolmogorov-Smirnov test) without significant changes in axon calibre. (**I**) Comparison of counts of total myelinated axons and axons myelinated by oligodendrocytes in vehicle control, tamoxifen control and conNrg1 animals. After ablation of Nrg1 the total number of myelinated axons is decreased (data are presented as mean ± SEM, *P < *0.05, one-way ANOVA, *post hoc* Tukey’s, *n = *3–4 animals/group) but the number of axons myelinated by oligodendrocytes is not altered (Vh control = 475 ± 326, Tx control = 338 ± 184 and conNrg1 = 117 ± 60). (**J**) The total number of axons present in the dorsal column after spinal cord contusion remains similar in all groups (data are presented as mean ± SEM, one-way ANOVA, *post hoc* Tukey’s, *n = *3–4 animals/group).

We used electron microscopy to analyse at the ultrastructural level myelination in the dorsal columns of injured control and conNrg1 mice. We observed a dramatic reduction in the number of myelinated axons in the dorsal column in conNrg1 mice at 10 weeks post injury compared to control mice (Fig. 2A–C and D) and a corresponding increase in the percentage of large diameter axons (diameter >1 µm) that were unmyelinated (Fig. 2A–C and E). There was no difference in axon diameter between groups (Fig. 2H), excluding the possibility of exacerbated axonal swelling in mice lacking Nrg1. We also assessed the effects of Nrg1 ablation on remyelination by Schwann cells versus oligodendrocytes in the dorsal columns using standard morphological criteria to differentiate these cell types (Felts and Smith, 1996; Woodruff and Franklin, 1999) (Supplementary Fig. 6). Only a negligible number of axons were remyelinated by Schwann cells in the dorsal column of conNrg1 mutant animals (Fig. 2A–C and F). The few remyelinated axons in injured conNrg1 mice were predominantly remyelinated by oligodendrocytes, but a compensatory increase in oligodendrocyte remyelination was not observed (Fig. 2C and I). Furthermore, those axons that were remyelinated by oligodendrocytes in conNrg1 mutant animals had significantly thinner myelin sheaths, quantitatively measured by an increased G-ratio (Fig. 2A–C and G). These changes were consistently observed between different animals, demonstrating that this striking demyelination phenotype is caused by the lack of Nrg1. Total axon counts did not differ between groups indicating that disruption of Nrg1 signalling had no effect on axonal survival at 10 weeks after spinal contusion injury (Fig. 2J).

**Figure 3:**
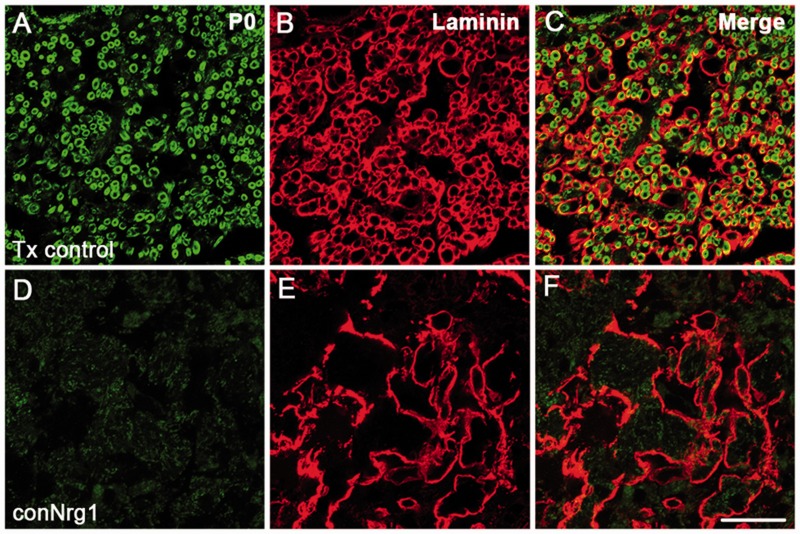
**Nrg1-dependent central axon remyelination after spinal cord injury is mediated by typical Schwann cells.** (**A**–**F**) Co-staining of basal lamina (laminin, red) and Schwann cell-associated myelin (P0, green) in tamoxifen control (Tx control, **A**–**C**) and Nrg1-ablated (conNrg1, **D**–**F**) mouse spinal cords 10 weeks after contusion injury reveals defined ring-like structures immunoreactive for laminin. These ring-like structures represent the typical basal lamina associated with Schwann cells, and are apparent in close proximity to P0-positive myelin rings in injured control spinal cord. No Schwann cell-associated myelin and only sparse and diffuse laminin staining is observed in spinal cords from conNrg1 mice. Scale bar = 25 µm. Images not using the red/green colour scheme are available in the Supplementary material.

To verify that remyelinating cells in the injured spinal cord were indeed ‘classic’ peripheral Schwann cells rather than type IV oligodendrocytes (which resemble peripheral Schwann cells but lack a basal lamina; Butt *et al.*, 1995; Butt and Berry, 2000), we assessed the presence of laminin, a key component of Schwann cell basal laminae. We observed prominent ring-like laminin-positive structures in close association with P0-positive myelin rings, indicating the presence of basal lamina around remyelinating PNS-like Schwann cells in the dorsal column of injured control animals (Fig. 3A–C). Strikingly, the complete absence of P0-positive myelin rings in the dorsal column of conNrg1 mice (Fig. 3D) was accompanied by an absence of laminin-positive rings (Fig. 3E), with only diffuse and disorganized laminin apparent in this region (Fig. 3D–F), which likely derives from other laminin-producing, but not remyelinating, cells.

Taken together, our detailed ultrastructural analysis confirms that Nrg1 is absolutely required for remyelination of dorsal column axons by PNS-like Schwann cells. Furthermore, these data imply that Nrg1 determines not only the myelinating potential of these cells, but also their occurrence in the injured spinal cord. Both electron microscopy data and immunohistochemistry support the conclusion that the appearance of ‘classic’ PNS-like Schwann cells in the injured spinal cord and the efficient remyelination of central axons occurs only in the presence of Nrg1.

### Nrg1 expression in normal and injured spinal cord

**Figure 4:**
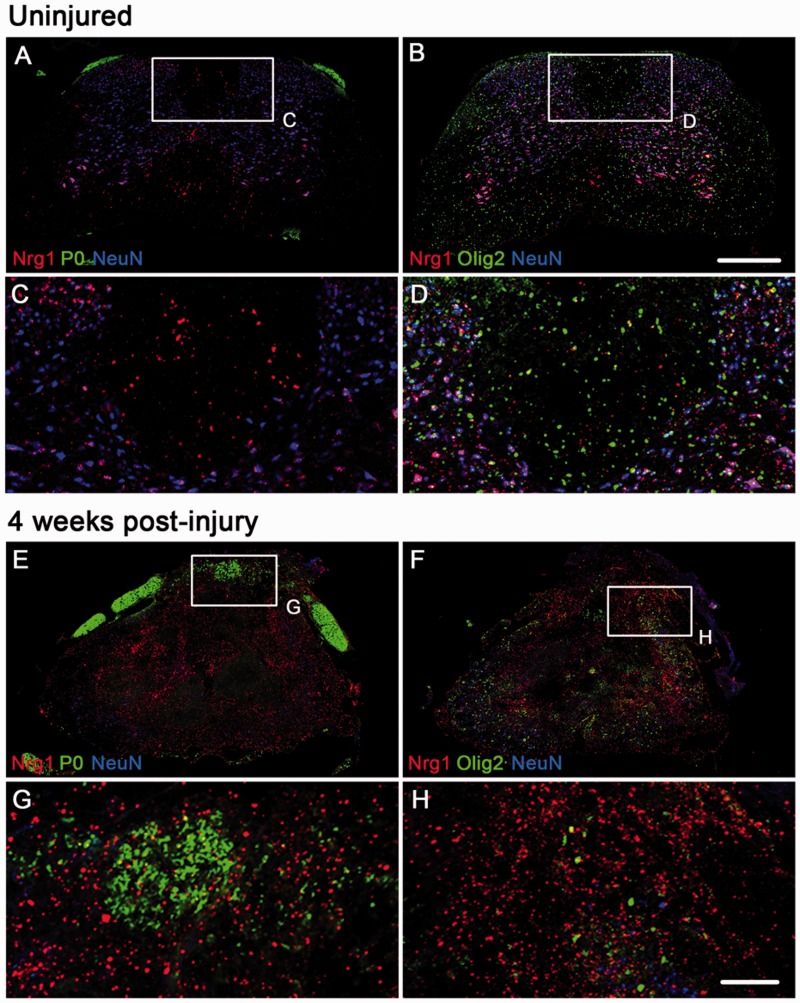
**Expression of Nrg1 before and after spinal cord injury.** Fluorescent *in situ* hybridization using a pan-Nrg1 probe (red) co-stained with markers for neurons (NeuN, blue), Schwann cell associated myelin (P0, green, **A**, **C**, **E**, **G**) and oligodendrocytes (Olig2, green, **B**, **D**, **F** and **H**). In the uninjured spinal cord (**A**–**D**) Nrg1 labelling can be seen within neurons. The highest level of expression is seen in motor neurons within the ventral horn; however Nrg1 is also expressed by neurons of the dorsal horn. In the uninjured state there is no co-localization of Nrg with P0 (**A** and **C**) and very few oligodendrocytes express Nrg1 (**B** and **D**). Four weeks after spinal contusion injury, compact myelin, which is P0 immunoreactive, can be seen within the dorsal column and there is little co-localization with Nrg1 (**E** and **G**). Similarly, few Olig2 immunoreactive profiles express Nrg1 (**F** and **H**). Scale bars = 250 µm (**B**); 100 µm (**H**). Images not using the red/green colour scheme are available in the Supplementary material.

To determine the expression pattern of Nrg1 before and after injury and the possible cellular source of neuregulin, we performed fluorescence *in situ* hybridization for Nrg1 using a pan-Nrg1 probe combined with double immunohistochemistry for P0 and NeuN or Olig2 and NeuN (Fig. 4) in uninjured wild-type mice (Fig. 4A–D) and contused wild-type mice at 4 weeks post-injury (Fig. 4E–H). As predicted from the literature (Corfas *et al.*, 1995; Meyer *et al.*, 1997), the majority of Nrg1 expression in uninjured spinal cords was neuronal, with high co-localization of Nrg1 in the cytoplasm of numerous NeuN-positive spinal neurons (Fig. 4A and B). Nrg1 was also apparent, although less abundant, in spinal cord white matter; this was not co-localized with P0, either in uninjured spinal cord where P0 was restricted to the peripheral dorsal roots (Fig. 4A and C), or injured spinal cord where P0 was also apparent in the spinal cord dorsal column (Fig. 4E and G). There was also little co-localization of Nrg1 with Olig 2 in the uninjured (Fig. 4B and D) or injured (Fig. 4F and H) spinal cord. Nrg1 is therefore unlikely to be having autocrine actions in Schwann cells or oligodendrocytes within the spinal cord. The majority of Nrg1 influencing Schwann cell remyelination is likely to be derived either from spinal neurons or axons projecting through the dorsal column: large diameter myelinated sensory neurons are known to express Nrg1 (particularly type III Nrg1) at a high level in adulthood (Bermingham-McDonogh *et al.*, 1997; Fricker *et al.*, 2009).

**Figure 5:**
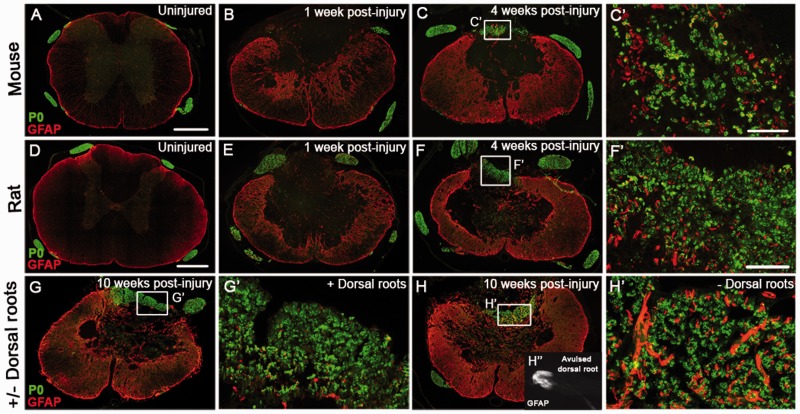
**Schwann cell-mediated remyelination in mouse and rat spinal cords follows the same time course and is unhindered by avulsion of multiple dorsal roots at and adjacent to the injury site.** (**A**–**C**) Co-staining of astrocytes (GFAP, red) and Schwann cell-associated myelin (P0, green), shows the time course of the appearance of Schwann cell myelin the injured mouse spinal course. Peripheral myelin (P0) is not apparent in uninjured mouse spinal cord (**A**) or at 1 week post-injury (**B**), but is present in the dorsal columns by 4 weeks after injury (**C**). **C’** shows a high magnification of the boxed area in panel **C**. (**D**–**F**) Co-staining of astrocytes (GFAP, red) and Schwann cell-associated myelin (P0, green), shows the time course of the appearance of Schwann cell myelin the injured rat spinal course. Peripheral myelin (P0) is not apparent in uninjured rat spinal cord (**D**) or at 1 week post-injury (**E**), but is present by 4 weeks after injury (**F**). **F’** shows high magnification of the boxed area in panel **F**. (**G** and **H**) At 10 weeks post-injury Schwann cell-mediated remyelination is apparent after spinal contusion injury irrespective of presence (**G**) or absence (**H**) of dorsal roots, one of the main possible peripheral sources of the Schwann cells. **G’** and **H’** show high magnifications of boxed areas in panels **G** and **H**. (**H’’**) Example of an avulsed dorsal root, which encompasses the entire rootlet including the dorsal root entry zone that is visualized by staining of the astroglial marker GFAP. Scale bars = 250 µm (**A** and **D**); 50 µm (**C’** and **F’**). Images not using the red/green colour scheme are available in the Supplementary material.

We also assessed mRNA expression of different Nrg1 isoforms and ErbB receptors, to determine whether spinal contusion injury alters the expression of components of the Nrg1-ErbB signalling pathway (Supplementary Fig. 7 and Supplementary Table 1), and determined how this correlates with remyelination after spinal cord injury. In particular, we assessed expression of Nrg1 isoforms types I and II (containing Ig-like domains) and type III (containing a cysteine-rich domain). Nrg1 type I expression within the spinal cord progressively increased at Weeks 1 and 4 post-injury (Supplementary Fig. 7A). In contrast, Nrg1 type II and type III expression had significantly decreased at 1 week post-injury, and recovered to ∼50% of the naïve levels 4 weeks post-injury (Supplementary Fig. 7A). These changes in Nrg1 isoform expression after injury were restricted to the spinal cord and not observed in peripheral dorsal root ganglia, where Nrg1 types I and II were unchanged and type III showed only a small transient decrease (Supplementary Fig. 8). Typically, Nrg1 exerts its effects via interaction with ErbB tyrosine kinase receptors (Birchmeier and Nave, 2008; Mei and Nave, 2014). Expression of ErbB3 and ErbB4 in the spinal cord followed a similar pattern to that of Nrg1 types II and III, and was significantly reduced at 1 week post-injury, with a subsequent partial recovery (Supplementary Fig. 7B). ErbB2 expression was increased at 4 weeks post-injury (Supplementary Fig. 7B). Thus, changes in expression of Nrg1 signalling components after injury appear to be consistent with the timing of Schwann cell-mediated remyelination in the spinal cord. Thus, when P0-positive Schwann cell-derived myelin is abundant in the dorsal columns (at 4 weeks, but not 1 week, post-injury; Fig. 5A–F) the expression of type III Nrg1 and ErbB3/4 has recovered and the expression of type I Nrg1 and erbB2 has increased.

### The majority of Schwann cells that remyelinate injured spinal axons have a CNS origin

The origin of remyelinating Schwann cells in CNS pathologies has been debated. To investigate the origin of the remyelinating Schwann cells after spinal cord injury, we removed peripheral input into the spinal cord with bilateral removal of multiple dorsal roots directly adjacent and feeding into the injury site. We used rats, rather than mice for these studies, due to the complexity of the surgery, after first determining that the time course of Schwann cell-mediated remyelination in the dorsal columns was the same in both species (Fig. 5A–F and James *et al.*, 2011). Efficient removal of the entire dorsal root and dorsal root entry zone was confirmed by the presence of a GFAP-positive astrocytic ‘cap’ on the avulsed root (Fig. 5H’’). No matter whether the roots were intact or removed, Schwann cell-derived myelin was apparent in the injured dorsal columns (Fig. 5G and H). Quantification revealed a small reduction in the amount of P0 myelin in the dorsal columns after multiple root avulsion, although this did not reach significance (Supplementary Fig. 9), indicating that the majority of the Schwann cell myelin in the dorsal columns derives from a central source, but at least some of the remyelinating Schwann cells may be peripherally derived. Alternatively, the amount of Schwann cell-mediated central remyelination may have been altered by changes in astrogliosis following multiple dorsal root removal, in line with recent findings (Monteiro de Castro *et al.*, 2015).

**Figure 6:**
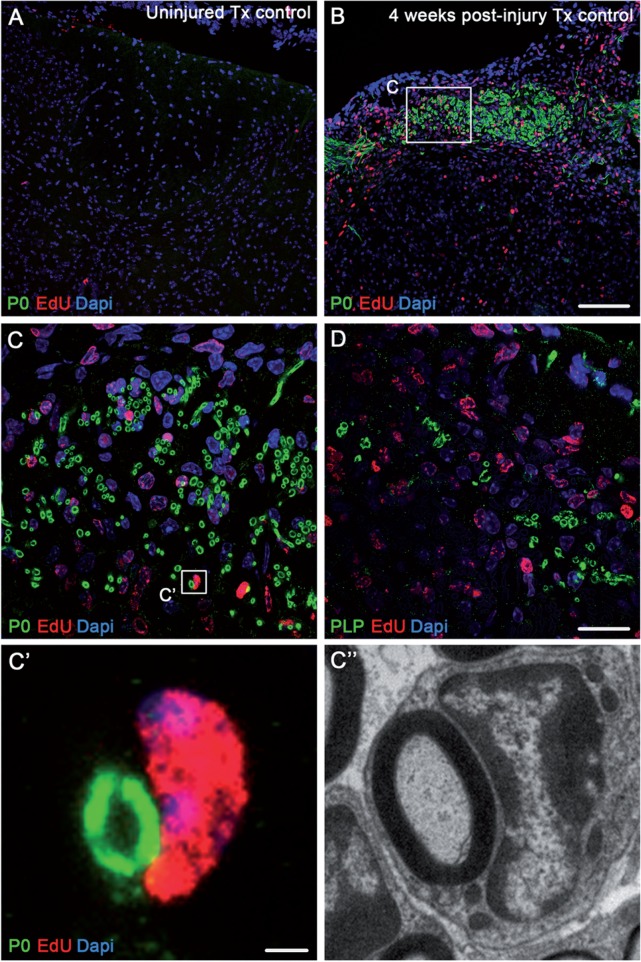
**Centrally remyelinating Schwann cells are produced *de novo* in the injured spinal cord.** (**A** and **B**) Immunohistochemical staining for Schwann cell myelin (P0, green), nuclear EdU (red) and DAPI (blue) shows abundant P0-positive myelin rings in close apposition with EdU-positive cell nuclei 4 weeks after contusion injury (**B**) but not in control uninjured spinal cords (**A**). (**C**) High magnification image showing the boxed area in **B**, demonstrating remyelinating EdU-positive Schwann cells in association with P0-positive Schwann cell-derived myelin. (**D**) High magnification image of dorsal column axons associated with central PLP-positive myelin. (**C’** and **C’’**) High magnification image showing the boxed area in **C**. Co-labelling clearly reveals direct apposition of a EdU-positive Schwann cell with a P0-positive myelin ring (**C’**) alongside an electron microscopic comparison of a Schwann cell in the dorsal columns that has remyelinated a CNS axon (**C’’**). Scale bars = 100 µm (**B**); 20 µm (**D**); 2 µm (**C’**). Images not using the red/green colour scheme are available in the Supplementary material.

To further determine whether remyelinating Schwann cells originate from a central source, we administered EdU to mice following spinal contusion and assessed for the presence of newly dividing cells in the spinal cord at 4 weeks post-injury (a time point when myelinating Schwann cells are present in the dorsal columns; Fig. 5C and F). Co-localization of EdU with P0 in spinal sections from these animals (Fig. 6) revealed numerous EdU-positive cells directly adjacent to P0-positive Schwann cell myelin rings (Fig. 6B and C), providing evidence that myelinating Schwann cells were newly generated endogenous precursor cells. We observed a striking comparison between the histological observations of EdU positive nuclei in close apposition to P0-positive Schwann cell myelin (Fig 6C’) and the electron microscopy observations of a one-to-one relationship of a Schwann cell remyelinating a central axon in the dorsal columns (Fig 6C’’). Co-localization of EdU with PLP revealed far fewer oligodendrocyte-derived myelin rings in the injured dorsal columns (Fig. 6D).

These data provide evidence that peripherally invading Schwann cells do not play a significant role in remyelination of spinal axons after spinal cord injury. Rather, the majority of these remyelinating Schwann cells derive from a CNS origin and are the major contributor to this endogenous repair mechanism. Our results suggest that Nrg1 is the molecular switch that drives precursor cells residing in the spinal cord to differentiate into PNS-like Schwann cells and to remyelinate spinal axons.

### Immunoglobulin-containing isoforms of Nrg1 are dispensable for Schwann cell remyelination

**Figure 7:**
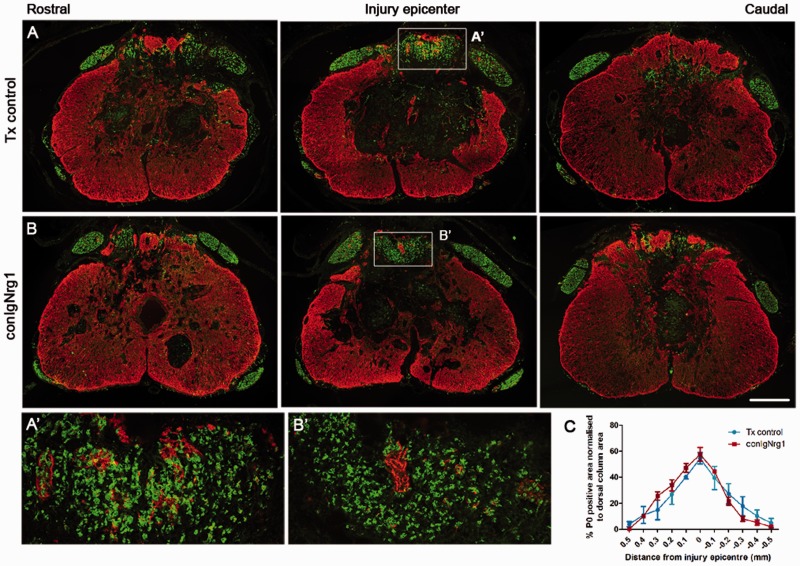
**Ablation of Ig-containing isoforms of Nrg1 (Nrg1 types 1 and 2) does not interfere with Schwann cell-mediated remyelination after spinal contusion injury.** (**A** and **B**) Co-staining of astrocytes (GFAP, red) and Schwann cell-associated myelin (P0, green) shows appearance of Schwann cell myelin the injured mouse spinal cord of tamoxifen control (Tx control, **A**) and IgNrg1-ablated mice (conIgNrg1, **B**). Ten weeks after contusion injury, Schwann cell-associated myelin (P0) is abundant in the dorsal column of spinal cords from both control animals and mice lacking the IgNrg1 isoforms, being particularly abundant in the lesion epicentre. (**A’** and **B’**) High magnification of boxed areas in **A** and **B**. (**C**) Quantification of P0-positive area in sections spanning the rostrocaudal axis of the injury site reveals similar levels in conIgNrg1 and control mice (^ns^*P* > 0.05, two-way ANOVA, *post hoc* Bonferroni, *n = *4 animals/group). Scale bars = 250 µm (**B**); 50 µm (**B’**). Images not using the red/green colour scheme are available in the Supplementary material.

Given the complex changes in Nrg1 isoform expression after spinal cord injury, we used isoform-specific mutant mice to distinguish functions of particular Nrg1 isoforms in spinal axon remyelination and recovery of locomotor function. To do this we used a strain of mice in which Ig domain-containing Nrg1 isoforms (Nrg1 types I and II) are ablated after tamoxifen treatment (conIgNrg1 mice), but the type III Nrg1 isoform remains intact (Cheret *et al.*, 2013). We first confirmed that after tamoxifen treatment, levels of IgNrg1 transcripts in the thoracic spinal cord and in dorsal root ganglia were significantly reduced when compared to tamoxifen-treated control littermates not expressing Cre (Supplementary Fig. 10A and B). Expression of type III Nrg1 was unchanged (Supplementary Fig. 10C and D). To analyse remyelination in these animals, we assessed the expression of Schwann cell-derived myelin throughout the lesion site after injury. P0 immunohistochemistry at 10 weeks after contusive spinal cord injury revealed that Schwann cell-mediated axonal remyelination is indistinguishable between tamoxifen-treated control and conIgNrg1 animals, with a similar rostrocaudal spread of P0 expression observed in the dorsal column (Fig. 7A–C). Schwann cell-associated myelin was most abundant at the lesion epicentre where ∼60% of the dorsal column area expressed peripheral myelin (Fig. 7C). This is therefore in stark contrast to conNrg1 animals in which all Nrg1 isoforms are ablated and suggests that type I and II isoforms (containing the Ig domain) are dispensable for central remyelination after spinal cord injury. Thus, the presence of the type III Nrg1 isoform suffices to trigger the remyelination process.

### Conditional ablation of Nrg1 has a significant impact on the level of spontaneous locomotor recovery after spinal cord injury

**Figure 8:**
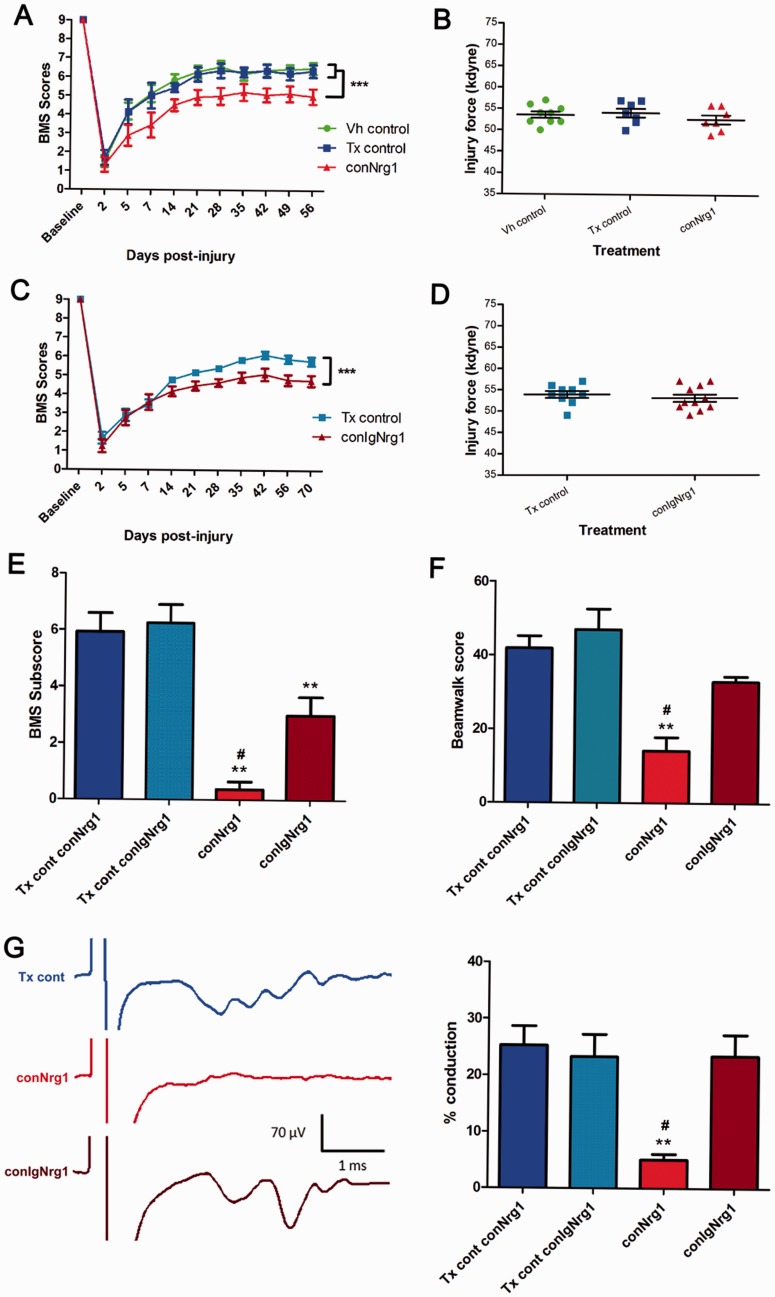
**Ablation of Nrg1 leads to impaired spontaneous functional recovery after spinal contusion injury.** Functional recovery assessed by BMS open field locomotion scores in conNrg1 (**A**) and conIgNrg1 (**C**) mice show a similar initial deficit in all groups acutely after spinal contusion injury. BMS scores gradually improve over the first few weeks and begin to plateau ∼3 weeks post-injury. Spontaneous functional recovery is significantly impaired in both conNrg1 (**A**) and conIgNrg1 (**C**) mutant mice, compared to vehicle and tamoxifen controls. Baseline BMS scores were not different between groups. Data are presented as mean ± SEM. (^***^P < 0.001, two-way ANOVA, *post hoc* Bonferroni, *n = *9–11 animals/group). (**B** and **D**) Contusion impact data showing the actual force applied to individual mice was within 10% of the intended force of 50 kdyne and mean values for each group were not significantly different (*P* > 0.05; one-way ANOVA), confirming that any group differences were not due to differences in the impact force during surgery. (**E**) BMS subscores reveal significantly reduced functional recovery both in conNrg1 and conIgNrg1 animals compared to controls at 8 weeks post-injury. However, conNrg1 mice were significantly more impaired than conIgNrg1 mice in areas including stepping frequency, coordination, paw position, trunk stability, and tail position. (**F**) Beam-walking scores reveal reduced beam-walking performance both in conNrg1 and conIgNrg1 animals compared to controls at 8 weeks post-injury. However, conNrg1 mice were significantly more impaired than conIgNrg1 mice. Data are presented as mean ± SEM (^**^P < 0.01, **^#^***P < *0.05, one-way ANOVA, *post hoc* Tukey’s, *n = *6–9 animals/group). *Significantly different to tamoxifen controls; **^#^**significantly different to conIgNrg1. (**G**) *In vivo* electrophysiological recordings assessing dorsal column function. Example traces (each averaged from 16 raw traces) show conduction through the lesion site in control, conNrg1 and conIgNrg1 animals (the representative control trace is taken from a conNrg1 tamoxifen control mouse). In control and conIgNrg1 injured animals stimulation artefacts, which have been cropped on the *x*-axis to allow appropriate scaling, were followed by evoked afferent activity at a latency of ∼1.5 ms, whereas little or no activity was evoked in conNrg1 animals. All stimulation was supramaximal. Quantification of the percentage of axons capable of conducting through the contusion site confirmed significant levels of dorsal column function in injured controls and conIgNrg1 mice at 10 weeks post-injury (when significant Schwann cell-mediated remyelination of the dorsal columns is apparent), which was dramatically reduced in conNrg1 animals (where Schwann cell-mediated remyelination is absent). Data are presented as mean ± SEM (^**^P < 0.01, **^#^***P < *0.05, one-way ANOVA, *post hoc* Tukey’s, *n = *5–6 animals/group); ^**^significantly different to tamoxifen controls; **^#^**significantly different to conIgNrg1.

To evaluate the functional consequences of Nrg1 ablation we first assessed gross locomotor function in conNrg1 and conIgNrg1 mice using the BMS open field hindlimb locomotor scale (Fig. 8A–D; BMS behaviour displayed separately as these were separate studies). In the conNrg1 study (Fig. 8A and B), no differences in baseline BMS ratings were detected in uninjured animals, regardless of whether Nrg1 was present or ablated. However, following spinal cord contusion Nrg1 ablation was associated with significant long-term impaired locomotor recovery (Fig. 8A and Supplementary Videos 1 and 2). Furthermore, we observed impaired paw rotation and forelimb-hindlimb coordination post-injury in conNrg1 mice compared to control mice, i.e. processes that require proprioceptive control mediated by dorsal column axons (Kanagal and Muir, 2008). However, it should be noted that the impact of Nrg1 ablation on spontaneous locomotor recovery started to emerge during the first 2 weeks after injury, a time where significant remyelination in the dorsal columns is not yet apparent following spinal contusion injury (James *et al.*, 2011). Therefore, in addition to the deficient remyelination, other mechanisms might also contribute to the changed locomotor recovery, such as deficient muscle spindle feedback, which has recently been shown to be important for locomotor recovery after spinal cord injury (Takeoka *et al.*, 2014). Indeed, Nrg1 is an important signal for muscle spindle maintenance and muscle spindles atrophy after Nrg1 ablation in the adult (Cheret *et al.*, 2013). We then assessed BMS scores in conIgNrg1 mice (Fig. 8C and D), where Schwann cell-mediated remyelination remains intact. Interestingly, in spite of preserved spontaneous remyelination, locomotor recovery was also impaired in conIgNrg1 mice compared to control injured animals (Fig. 8C). In general, animals showed impairments in coordination similar to, but not as extensive as, the animals lacking all Nrg1 isoforms (*cf*. Fig. 8A and C).

To further elucidate the contribution of Nrg1 to functional repair after spinal cord injury, we performed additional functional studies in new cohorts of conNrg1 and conIgNrg1 mice to enable side-by-side comparisons in a number of additional behavioural outcomes (Fig. 8E and F). The 12-point BMS subscore scale, which further delineates recovery of specific locomotor features that may not be apparent in the overall BMS score (Basso *et al.*, 2006), revealed that while both cohorts were significantly impaired in comparison to control injured mice, the conNrg1 mice were also significantly worse than conIgNrg1mice at 8 weeks post-injury (^#^*P < *0.05, conNrg1 versus conIgNrg1; average score 0.4 ± 0.26 and 3 ± 0.65, respectively; Fig. 8E) on aspects of behaviour related to stepping frequency, coordination, paw position, trunk stability, and tail position. Furthermore, functional performance was also assessed on the beam walking task, which requires co-ordination, balance and proprioception (functions that are directly related to dorsal column function; Kanagal and Muir, 2008). Again, conNrg1 mice performed significantly worse than injured conIgNrg1mice on the beam walking task (**^#^***P < *0.05, conNrg1 versus conIgNrg1; average score 14 ± 3.7 and 33 ± 1.5, respectively; Fig. 8F). These data clearly demonstrate that there was significantly worse functional outcome in conNrg1 mice (with the profound dorsal column demyelinating phenotype) than in conIgNrg1 mice (where Schwann cell-mediated remyelination was not altered), particularly in tasks that require dorsal column function.

### Conditional ablation of Nrg1 leads to conduction failure in dorsal column axons after spinal cord injury

Although behavioural differences suggest an association between the profound demyelinating phenotype in conNrg1 mice and worse functional outcome, a more robust measure for determining the functional importance of Schwann cell-mediated remyelination after spinal cord injury and how this is compromised by Nrg1 ablation is to assess the electrophysiological properties of dorsal column axons in control and Nrg1 mutant mice following spinal contusion. In a protocol adapted from a previous method in rats (James et al., 2011), terminal electrophysiological recordings taken from the sural nerve revealed a clear difference in the ability of long distance sensory fibres travelling in the dorsal columns to conduct through the T10 spinal contusion site (Fig. 8G). Sensory dorsal column fibres were activated antidromically by firstly stimulating 5 mm caudal of the lesion site. Recordings taken from the sural nerve whilst stimulating supramaximally therefore represented the function of the intact portion of this pathway. Stimulation was then moved 5 mm rostral of the lesion site to determine what extent of this function remained through the lesion site. Recordings taken when stimulating at each site were averaged and the amplitude of the response in the sural nerve when stimulating rostral to the lesion was calculated as a percentage of the response evoked when stimulating caudally. In both of the tamoxifen control cohorts and the con IgNrg1 cohort, all animals displayed obvious evoked activity in the sural nerve when stimulating rostrally; in contrast to this, the majority of conNrg1 animals (four of five) displayed no detectable evoked activity in the sural nerve, and in the single animal which displayed some activity this was minimal (representative traces in Fig. 8G). Quantification revealed that this difference in the conduction of dorsal column fibres was highly significant (graph in Fig. 8G; **P < 0.01, ^#^*P < *0.05, one-way ANOVA, Tukey’s *post hoc*), with the conNrg1 group having a mean conduction of only 5.05% ± 1.03 and all other groups having conduction of ∼25% (conNrg1 tamoxifen control: 25.27% ±3.42; IgNrg1 tamoxifen control: 23.26% ± 3.96; conIgNrg1: 23.45% ± 3.77). These data provide robust in vivo evidence firstly, that dorsal column axons remyelinated by Schwann cells after spinal cord injury are functional and secondly, that the profound demyelinating phenotype observed in the dorsal columns of conNrg1 knockout mice, and the associated functional impairment, corresponds to axonal conduction failure.

## Discussion

Our data show that Nrg1 is a key regulator of remyelination of CNS axons by PNS-like Schwann cells after spinal cord injury. The majority of these centrally occurring Schwann cells are derived from the CNS, and arise most likely through (trans)-differentiation of precursor cells that reside in the spinal cord. Furthermore, we show that interference with Nrg1 signalling significantly reduces the degree of spontaneous locomotor recovery after injury. Using Nrg1 isoform-specific mutant mice, we found that IgNrg1 isoforms are dispensable for Schwann cell myelination, indicating that type III (cysteine-rich domain containing) isoforms have a critical role in this repair process. Although IgNrg1 isoforms do not mediate Schwann cell remyelination, they contribute to recovery of locomotor function. We assign this to the role of IgNrg1 in muscle spindle maintenance. The functional impairment in those animals in which all Nrg1 isoforms are inactivated is greater than in the IgNrg1 specific mutants, particularly in measures relating to dorsal column function corresponding with profound conduction block observed on electrophysiological assessment of sensory axons projecting through the injury site within the dorsal column. Our data emphasize the distinct roles of Nrg1 isoforms in intrinsic repair processes after traumatic spinal cord injury. Manipulating Nrg1 signalling might thus lead to novel therapeutic strategies, or form part of a combinatorial therapy, for enhancing repair after spinal cord injury.

The injured CNS has some intrinsic capacity to spontaneously repair. Understanding the mechanisms that regulate or restrict these natural regenerative processes may lead to novel clinical paradigms that enhance spontaneous recovery after spinal cord injury or augment the efficacy of existing therapeutic approaches. Experimental contusive spinal cord injury resembles many human spinal cord injuries (Bunge *et al.*, 1993; Kakulas, 1999; Norenberg *et al.*, 2004). An important feature of contusion-type injuries is the sparing of a peripheral rim of tissue that contains viable axons. Demyelination is a key pathological characteristic of surviving axons that limit their ability to function properly, as these focally demyelinated axons are unable to efficiently conduct action potentials (Koles and Rasminsky, 1972; Nashmi and Fehlings, 2001). These surviving axons remain non-functional but represent an important therapeutic target (James et al., 2011). Remyelination and restoration of conduction may lead to detectable functional improvement (Smith *et al.*, 1979; Waxman *et al.*, 1994). Robust spontaneous myelin repair can occur, which is mediated by both immature oligodendrocytes and Schwann cells (Harrison *et al.*, 1975; McDonald, 1975). Schwann cell-mediated remyelination in the CNS has been reported after spinal cord injury in many species including rodents (Beattie *et al.*, 1997; James *et al.*, 2011), cats (Bunge *et al.*, 1961; Blight and Young, 1989), monkeys (Bresnahan, 1978) and humans (Bunge *et al.*, 1993; Bruce *et al.*, 2000; Guest *et al.*, 2005). It has also been observed in other central pathologies such as multiple sclerosis (Ghatak *et al.*, 1973; Itoyama *et al.*, 1985) and compressive spondylotic myelopathy (Fehlings and Skaf, 1998). Moreover, central axons that are remyelinated by Schwann cells can regain function (Blight and Young, 1989). Thus, the phenomenon of Schwann cell-mediated remyelination of central axons is well known, but the molecular mechanism of this spontaneous regenerative event has remained obscure. We now show for the first time that a lack of Nrg1 entirely prevents remyelination of spinal axons by PNS-like Schwann cells after spinal cord injury. We observed abundant peripheral myelin in the dorsal columns of the injured spinal cord, which was completely absent when Nrg1 is lacking. This striking phenotype was confirmed by detailed ultrastructural analysis, which demonstrated a complete lack of PNS-like Schwann cells in the central dorsal columns accompanied by remyelination failure. Oligodendrocytes, which myelinate different calibre axons in the mammalian CNS, are heterogenous. Type IV oligodendrocytes morphologically resemble Schwann cells that myelinate PNS axons but lack the characteristic basal laminae of Schwann cells (Cornbrooks *et al.*, 1983; Butt *et al.*, 1995). We observed clear laminin immunoreactivity surrounding P0-positive myelin rings in the injured spinal cord, demonstrating that the cells responsible for remyelination contain a basal lamina. Thus, remyelinating cells are typical Schwann cells rather than type IV oligodendrocytes. This ring-like association of laminin-positive basal lamina with Schwann cell derived P0-positive myelin was entirely absent in mice lacking Nrg1, which is consistent with the absence of Schwann cells. However, diffuse laminin staining was still observed in the injured spinal cord from Nrg1-ablated animals, which is likely to be derived from other cells present at the injury site such as fibroblasts or vascular components (Elkhal *et al.*, 2004; Soderblom *et al.*, 2013).

In a number of other CNS disorders (such as stroke), Nrg1 has been shown to have neuroprotective effects (Croslan *et al.*, 2008; Iaci *et al.*, 2010). Therefore we performed axonal counts in the dorsal columns to determine the effects of Nrg1 on axonal survival after spinal cord injury. Although we observed no differences between injured controls and conNrg1 mice in the total number of surviving axons 10 weeks after spinal cord injury, it is conceivable that axons that remain persistently demyelinated in Nrg1-ablated animals would be more susceptible to axonal loss and degeneration at later time points and this would likely exacerbate functional impairment, as has been observed in other CNS pathologies (Bjartmar *et al.*, 2000; Lovas *et al.*, 2000; McGavern *et al.*, 2000; Lee *et al.*, 2012).

Despite significant spontaneous remyelination, some axons remain chronically demyelinated after spinal cord injury (Blight and Decrescito, 1986; Waxman, 1989; Bunge *et al.*, 1993; Crowe *et al.*, 1997; Guest *et al.*, 2005). Recent work showed that the time course of demyelination and remyelination corresponded to functional changes, and demonstrated that a population of chronically demyelinated axons retain the potential to conduct (James *et al.*, 2011). Our data confirm that demyelinated axons persist into the chronic period after contusive injury, but a large population of axons are robustly remyelinated by PNS-like Schwann cells. The importance of such remyelination is emphasized by the profound conduction failure in sensory axons projecting through the dorsal columns in conNrg1 mice. Our findings also highlight the poor capacity of oligodendrocytes to mediate remyelination after spinal cord injury. In the presence or absence of Nrg1, only a few axons were surrounded by abnormally thin oligodendrocyte-produced myelin. The lack of compensatory remyelination by oligodendrocytes in Nrg1-ablated mice was also evident by the unchanged expression of PLP-positive central myelin and the oligodendrocyte-specific transcription factor Olig2 at time points when axons are normally robustly remyelinated by Schwann cells (Brinkmann *et al.*, 2008; Makinodan *et al.*, 2012). Nrg1 ablation might have potentially influenced oligodendrocyte-mediated remyelination. However, our analysis presented here, as well as previous work, indicates that oligodendrocyte-mediated myelination/remyelination is not absolutely dependent on Nrg1 (Brinkmann *et al.*, 2008; Lundgaard *et al.*, 2013). Schwann cell remyelination of central axons after injury appears to be limited to the dorsal column region of the spinal cord, as we observed here, while other lateral and ventral white matter tracts remain persistently demyelinated, or are remyelinated predominantly by oligodendrocytes derived from precursor cells (Siegenthaler *et al.*, 2007). Here we focused on the effects of Nrg1 on demyelination and remyelination in the dorsal column. It remains to be seen whether Nrg1 also affects remyelination in other white matter tracts, and whether increasing Nrg1 expression could increase remyelination in other regions of the spinal cord.

The cues that govern the occurrence of endogenous PNS-like Schwann cells in the injured CNS are unknown. Schwann cells have been suggested to migrate from peripheral sources such as the dorsal roots into the spinal cord after an injury (Sims *et al.*, 1998; Jasmin *et al.*, 2000; Perlin *et al.*, 2011), or to originate from endogenous oligodendrocyte precursor cells in the spinal cord (Zawadzka *et al.*, 2010). The predominant localization of remyelinating Schwann cells in the dorsal columns, close to the dorsal root entry zone PNS/CNS interface, logically favours a peripheral source. However, here we demonstrate for the first time that removing the peripheral source of Schwann cells by multiple dorsal root removal had little impact on Schwann cell-mediated remyelination of dorsal column axons. The small reduction in P0 following multiple root removal indicates that a minor proportion of the remyelinating Schwann cells may derive from the periphery. However, as the majority of P0 myelin expression still remained in the dorsal columns after removal of peripheral input, this supports the notion that the majority of these remyelinating Schwann cells derive from the spinal cord, possibly from spinal cord- intrinsic progenitor populations. A potential mechanism is a spontaneous, injury induced (trans)-differentiation of endogenous oligodendrocyte precursors into Schwann cells, which is consistent with evidence obtained in models of focal demyelination, where fate mapping of progenitor cells in the adult spinal cord revealed a previously unappreciated capacity of CNS precursors to generate myelinating Schwann cells (Zawadzka *et al.*, 2010). Our data demonstrate that this phenomenon also occurs after traumatic spinal cord injury, with the observation of EdU and P0 co-expression providing direct evidence that myelinating Schwann cells in the contused spinal cord were newly generated cells. This is also in line with recent lineage tracing studies, which used genetic reporters of endogenous precursor cells to demonstrate that remyelinating Schwann cells after spinal contusion are primarily central in origin (Assinck *et al.*, 2015). Together, these findings provide strong evidence for a central origin of remyelinating Schwann cells following clinically relevant traumatic spinal contusion injury.

Concomitantly with the initiation of Schwann cell-mediated remyelination of dorsal column axons, we noted complex changes in expression of Nrg1 isoforms and their receptors. Analysis of isoform-specific mRNA expression showed parallels to the changes observed within peripheral nerve following injury. In particular, type I *Nrg1* was upregulated (Cohen *et al.*, 1992; Carroll *et al.*, 1997; Stassart *et al.*, 2013) and the expression of type II and III *Nrg1* isoforms were reduced 1 week after injury, but expression began to recover towards naïve levels 4 weeks after injury. Lumbar dorsal root ganglia (containing the cell bodies of dorsal column projecting axons) did not display major changes in *Nrg1* isoform expression. *Erbb3* and *Erbb4* receptor mRNA within the spinal cord was reduced at Week 1 but largely recovered by Week 4 post-injury; *Erbb2* showed increased expression at Week 4 post-injury. Given that many cell types including neurons, glia (Mei and Nave, 2014) and immune cells (Calvo *et al.*, 2010) express these receptors, this dynamic pattern of expression is likely to reflect in addition to altered expression in individual cells the changed cellular composition that is influenced by cell death, acute inflammation and subsequent endogenous repair following spinal cord injury. In general, by 4 weeks post-injury the expression of Nrg1 signalling components is replenished or enhanced, and this coincides with progressive remyelination of demyelinated axons by Schwann cells in the injured spinal cord. Our *in situ* hybridization expression data indicated that the main source of Nrg1 influencing Schwann cell remyelination after spinal cord injury is likely to be derived from spinal neurons or axons projecting through the dorsal column, as Nrg1 was apparent in the dorsal columns but did not co-localize with markers of Schwann cell myelin or oligodendrocytes. This is consistent with previous work which has shown high levels of expression of Nrg1 (particularly type III *Nrg1*) in the large diameter myelinated sensory neurons (Bermingham-McDonogh *et al.*, 1997; Fricker *et al.*, 2009), whose axons project in the dorsal column.

Nrg1 isoforms signal in distinct fashions: Types I and II (containing Ig domains) are directly secreted or shed from the cell membrane while type III (containing a cysteine rich domain) typically remains tethered to the cell membrane to signal in a juxtacrine fashion, although further processing can release the EGF domain in certain contexts (Fleck *et al.*, 2013). In the PNS, type III Nrg1 is the key factor that regulates many aspects of Schwann cell development and function, including myelination, which is absolutely dependent on type III Nrg1 (Garratt *et al.*, 2000; Nave and Salzer, 2006; Mei and Nave, 2014). In contrast, although both type I and type III Nrg1 isoforms have been shown to influence central myelination, oligodendrocytes (unlike peripheral Schwann cells) have evolved a Nrg1-independent mechanism of myelination (Brinkmann *et al.*, 2008; Nave and Werner, 2014). Interestingly after peripheral nerve injury in adulthood there is evidence that both axonal (which is principally type III Nrg1) (Fricker *et al.*, 2011) and Schwann cell-derived type I Nrg1 contribute to Schwann cell remyelination (Stassart *et al.*, 2013). In contrast, we did not observe Nrg1 expression by Schwann cells within the injured spinal cord. We have taken advantage of isoform-specific mutant mice to investigate the relative contribution of the different isoforms in adulthood after CNS injury. Schwann cell-mediated remyelination of the dorsal columns was absent in mice lacking all active Nrg1 isoforms, while the lack of only IgNrg1 isoforms did not interfere with Schwann cell-mediated remyelination, emphasizing the role of type III Nrg1 in this repair process. The fact that IgNrg1 is dispensable for Schwann cell remyelination in the spinal cord is consistent with the previous finding that infusion of glial growth factor (Ig containing type II Nrg1 isoform) did not improve Schwann cell-mediated remyelination after a gliotoxic injury to the cerebellar peduncle (Penderis *et al.*, 2003). Unexpectedly, however, spontaneous locomotor recovery was significantly compromised irrespective of the presence or absence of type III Nrg1. This indicates that the functional deficits were not entirely due to the striking demyelination phenotype observed in mice lacking all Nrg1 isoforms. The importance of Ig-containing Nrg1 in the regulation of muscle spindle development and physiology has previously been demonstrated (Hippenmeyer *et al.*, 2002; Cheret *et al.*, 2013). Thus, the behavioural phenotype observed in our study may involve deficient muscle spindle-dependent sensory feedback to the spinal cord, which has recently been shown to be critical for basic locomotor recovery after spinal cord injury (Takeoka *et al.*, 2014). However, to elucidate further the contribution of dorsal column demyelination to the locomotor deficits, we performed further detailed functional assessments in additional cohorts of animals. We directly compared functional recovery in conNrg1 mice (in which all Nrg1 isoforms are inactivated) and in conIgNrg1 animals in which there is selective inactivation of Ig isoforms. Although both mutants showed functional deficits, these were most marked in the conNrg1 compared to conIgNrg1, emphasizing the role of Schwann cell remyelination within the dorsal column for optimal recovery. This was apparent both on the BMS subscale and the inclined beam walking task, which requires co-ordination, balance and proprioception—functions that relate to the dorsal column pathway, through which myelinated proprioceptive sensory afferents transit. To directly localize this deficit to the dorsal column we undertook electrophysiological assessment by antidromic stimulation of sural afferents (which are principally sensory) within the dorsal column rostral and caudal to the injury site. While afferents projecting through the injury site could be detected in control animals (the evoked compound sensory action potential recorded in the sural nerve following rostral stimulation was 25% of that following caudal stimulation) there was a profound deficit in conduction in the conNrg1 mice (which in most cases showed no conduction through the injury site). In contrast, the conIgNrg1 mice demonstrated impulse conduction which was no different to controls, consistent with the fact that Schwann cell remyelination was unchanged in these animals.

In summary, our data demonstrate that Nrg1 is absolutely essential for Schwann cell-mediated spinal axon remyelination following spinal cord injury. We provide evidence that these central Schwann cells derive from the injured spinal cord and are required for action potential conduction in axons passing through the injury site. IgNrg1 isoforms are dispensable for this process; however, compromised IgNrg1 signalling also significantly impairs spontaneous locomotor recovery after spinal cord injury, likely a consequence of its role in muscle spindle maintenance. However, mice in which all Nrg1 isoforms are inactivated show greater functional deficits than IgNrg1 specific mutants, emphasizing the importance of Schwann cell remyelination of dorsal column axons for optimal functional recovery following spinal cord injury. Our data provide novel evidence for the molecular mechanisms that govern a spontaneous endogenous regenerative event after traumatic spinal cord injury, which may lead to the development of new therapeutic strategies. Enhancing levels of Nrg1 may accelerate this remyelination process and/or prime the injury site to facilitate integration of transplanted cells and/or enhance their remyelinating capacity. Schwann cell-mediated remyelination of spinal axons is also observed in other pathologies such as multiple sclerosis in humans (Ghatak *et al.*, 1973; Itoyama *et al.*, 1985); as such, these data may be exploited to improve or facilitate this regenerative process following spinal cord injury or other pathologies where demyelination occurs.

## Supplementary Material

Supplementary Fig. 5

## Abbreviations

BMS: Basso Mouse Scale
IgNrg1: immunoglobulin-containing isoform of Nrg1
Nrg1: neuregulin 1
PNS: peripheral nervous system

## References

[aww039-B1] Assinck PL Duncan G Plemel J Lee M Liu J Bergles D, PDGFRα-positive progenitor cells form myelinating oligodendrocytes and Schwann cells following contusion spinal cord injury. Society for Neuroscience Abstract 2015, Program No. 338.03. Chicago; 2015.

[aww039-B2] Basso DM Fisher LC Anderson AJ Jakeman LB McTigue DM Popovich PG Basso Mouse Scale for locomotion detects differences in recovery after spinal cord injury in five common mouse strains. J Neurotrauma 2006; 23: 635–59. May; 16689667 10.1089/neu.2006.23.635

[aww039-B3] Beattie MS Bresnahan JC Komon J Tovar CA Van Meter M Anderson DK, Endogenous repair after spinal cord contusion injuries in the rat. Exp Neurol 1997; 148: 453–63.; 9417825 10.1006/exnr.1997.6695

[aww039-B4] Beattie MS Harrington AW Lee R Kim JY Boyce SL Longo FM, ProNGF induces p75-mediated death of oligodendrocytes following spinal cord injury. Neuron 2002; 36: 375–86. 12408842 10.1016/s0896-6273(02)01005-x PMC2681189

[aww039-B5] Bermingham-McDonogh O Xu YT Marchionni MA Scherer SS Neuregulin expression in PNS neurons: isoforms and regulation by target interactions. Mol Cell Neurosci 1997; 10: 184–95. 9532580 10.1006/mcne.1997.0654

[aww039-B6] Birchmeier C Nave KA Neuregulin-1, a key axonal signal that drives Schwann cell growth and differentiation. Glia 2008; 56: 1491–7. Nov 1; 18803318 10.1002/glia.20753

[aww039-B7] Bjartmar C Kidd G Mork S Rudick R Trapp BD Neurological disability correlates with spinal cord axonal loss and reduced N-acetyl aspartate in chronic multiple sclerosis patients. Ann Neurol 2000; 48: 893–901. 11117546

[aww039-B8] Black JA Waxman SG Smith KJ Remyelination of dorsal column axons by endogenous Schwann cells restores the normal pattern of Nav1.6 and Kv1.2 at nodes of Ranvier. Brain 2006; 129: 1319–29. 16537565 10.1093/brain/awl057

[aww039-B9] Blight AR Decrescito V Morphometric analysis of experimental spinal cord injury in the cat: the relation of injury intensity to survival of myelinated axons. Neuroscience 1986; 19: 321–41. Sep; 3785669 10.1016/0306-4522(86)90025-4

[aww039-B10] Blight AR Young W Central axons in injured cat spinal cord recover electrophysiological function following remyelination by Schwann cells. J Neurol Sci 1989; 91: 15–34. Jun; 2746287 10.1016/0022-510x(89)90073-7

[aww039-B11] Bresnahan JC An electron-microscopic analysis of axonal alterations following blunt contusion of the spinal cord of the rhesus monkey (Macaca mulatta). J Neurol Sci 1978; 37: 59–82. Jun; 99494 10.1016/0022-510x(78)90228-9

[aww039-B12] Brinkmann BG Agarwal A Sereda MW Garratt AN Muller T Wende H, Neuregulin-1/ErbB signaling serves distinct functions in myelination of the peripheral and central nervous system. Neuron 2008; 59: 581–95. 18760695 10.1016/j.neuron.2008.06.028 PMC2628490

[aww039-B13] Bruce JH Norenberg MD Kraydieh S Puckett W Marcillo A Dietrich D Schwannosis: role of gliosis and proteoglycan in human spinal cord injury. J Neurotrauma 2000; 17: 781–8. Sep; 11011818 10.1089/neu.2000.17.781

[aww039-B14] Bunge MB Bunge RP Ris H Ultrastructural study of remyelination in an experimental lesion in adult cat spinal cord. J Biophys Biochem Cytol 1961; 10: 67–94. May; 13688845 10.1083/jcb.10.1.67 PMC2225064

[aww039-B15] Bunge RP Bunge MB Rish H Electron microscopic study of demyelination in an experimentally induced lesion in adult cat spinal cord. J Biophys Biochem Cytol 1960; 7: 685–96. 13805917 10.1083/jcb.7.4.685 PMC2224890

[aww039-B16] Bunge RP Puckett WR Becerra JL Marcillo A Quencer RM Observations on the pathology of human spinal cord injury: a review and classification of 22 new cases with details from a case of chronic cord compression with extensive focal demyelination. Adv Neurol 1993; 59: 75–89. 8420126

[aww039-B17] Buss A Pech K Merkler D Kakulas BA Martin D Schoenen J, Sequential loss of myelin proteins during Wallerian degeneration in the human spinal cord. Brain 2005; 128: 356–64. Feb; 15634734 10.1093/brain/awh355

[aww039-B18] Butt AM Berry M Oligodendrocytes and the control of myelination in vivo: new insights from the rat anterior medullary velum. J Neurosci Res 2000; 59: 477–88. 10679786 10.1002/(SICI)1097-4547(20000215)59:4<477::AID-JNR2>3.0.CO;2-J

[aww039-B19] Butt AM Ibrahim M Ruge FM Berry M Biochemical subtypes of oligodendrocyte in the anterior medullary velum of the rat as revealed by the monoclonal antibody Rip. Glia 1995; 14: 185–97. 7591030 10.1002/glia.440140304

[aww039-B20] Calvo M Zhu N Tsantoulas C Ma Z Grist J Loeb JA, Neuregulin-ErbB signaling promotes microglial proliferation and chemotaxis contributing to microgliosis and pain after peripheral nerve injury. J Neurosci 2010; 30: 5437–50. 20392965 10.1523/JNEUROSCI.5169-09.2010 PMC2862659

[aww039-B21] Cannon B Sensation and loss. Nature 2013; 503: S2–3. 24227004 10.1038/503S2a

[aww039-B22] Carroll SL Miller ML Frohnert PW Kim SS Corbett JA Expression of neuregulins and their putative receptors, ErbB2 and ErbB3, is induced during Wallerian degeneration. J Neurosci 1997; 17: 1642–59. 9030624 10.1523/JNEUROSCI.17-05-01642.1997 PMC6573392

[aww039-B23] Cheret C Willem M Fricker FR Wende H Wulf-Goldenberg A Tahirovic S, Bace1 and Neuregulin-1 cooperate to control formation and maintenance of muscle spindles. EMBO J 2013; 32: 2015–28. 23792428 10.1038/emboj.2013.146 PMC3715864

[aww039-B24] Chevalier Z Kennedy P Sherlock O Spinal cord injury, coping and psychological adjustment: a literature review. Spinal Cord 2009; 47: 778–82. 19506568 10.1038/sc.2009.60

[aww039-B25] Cohen JA Yachnis AT Arai M Davis JG Scherer SS Expression of the neu proto-oncogene by Schwann cells during peripheral nerve development and Wallerian degeneration. J Neurosci Res 1992; 31: 622–34. 1374476 10.1002/jnr.490310406

[aww039-B26] Corfas G Rosen KM Aratake H Krauss R Fischbach GD Differential expression of ARIA isoforms in the rat brain. Neuron 1995; 14: 103–15. 7530017 10.1016/0896-6273(95)90244-9

[aww039-B27] Cornbrooks CJ Carey DJ McDonald JA Timpl R Bunge RP In vivo and in vitro observations on laminin production by Schwann cells. Proc Natl Acad Sci USA 1983; 80: 3850–4. Jun; 6344090 10.1073/pnas.80.12.3850 PMC394150

[aww039-B28] Croslan DR Schoell MC Ford GD Pulliam JV Gates A Clement CM, Neuroprotective effects of neuregulin-1 on B35 neuronal cells following ischemia. Brain Res 2008; 1210: 39–47. 18410912 10.1016/j.brainres.2008.02.059 PMC2442468

[aww039-B29] Crowe MJ Bresnahan JC Shuman SL Masters JN Beattie MS Apoptosis and delayed degeneration after spinal cord injury in rats and monkeys. Nat Med 1997; 3: 73–6. Jan; 8986744 10.1038/nm0197-73

[aww039-B30] Dietz V Fouad K Restoration of sensorimotor functions after spinal cord injury. Brain 2014; 137: 654–67. 24103913 10.1093/brain/awt262

[aww039-B31] Duncan ID Brower A Kondo Y Curlee JF Jr Schultz RD Extensive remyelination of the CNS leads to functional recovery. Proc Natl Acad Sci USA 2009; 106: 6832–6. 19342494 10.1073/pnas.0812500106 PMC2672502

[aww039-B32] Elkhal A Tunggal L Aumailley M Fibroblasts contribute to the deposition of laminin 5 in the extracellular matrix. Exp Cell Res 2004; 296: 223–30. 15149852 10.1016/j.yexcr.2004.02.020

[aww039-B33] Falls DL Neuregulins: functions, forms, and signaling strategies. Exp Cell Res 2003; 284: 14–30. 12648463 10.1016/s0014-4827(02)00102-7

[aww039-B34] Fawcett JW Curt A Steeves JD Coleman WP Tuszynski MH Lammertse D, Guidelines for the conduct of clinical trials for spinal cord injury as developed by the ICCP panel: spontaneous recovery after spinal cord injury and statistical power needed for therapeutic clinical trials. Spinal Cord 2007; 45: 190–205. 17179973 10.1038/sj.sc.3102007

[aww039-B35] Fehlings MG Skaf G A review of the pathophysiology of cervical spondylotic myelopathy with insights for potential novel mechanisms drawn from traumatic spinal cord injury. Spine 1998; 23: 2730–7. 9879098 10.1097/00007632-199812150-00012

[aww039-B36] Felts PA Smith KJ Conduction properties of central nerve fibers remyelinated by Schwann cells. Brain Res 1992; 574: 178–92. 1638392 10.1016/0006-8993(92)90815-q

[aww039-B37] Felts PA Smith KJ Blood-brain barrier permeability in astrocyte-free regions of the central nervous system remyelinated by Schwann cells. Neuroscience 1996; 75: 643–55. 8931026 10.1016/0306-4522(96)00282-5

[aww039-B38] Fitch MT Silver J CNS injury, glial scars, and inflammation: Inhibitory extracellular matrices and regeneration failure. Exp Neurol 2008; 209: 294–301. 17617407 10.1016/j.expneurol.2007.05.014 PMC2268907

[aww039-B39] Fleck D van Bebber F Colombo A Galante C Schwenk BM Rabe L, Dual cleavage of neuregulin 1 type III by BACE1 and ADAM17 liberates its EGF-like domain and allows paracrine signaling. J Neurosci 2013; 33: 7856–69. 23637177 10.1523/JNEUROSCI.3372-12.2013 PMC6618983

[aww039-B40] Franklin RJ Ffrench-Constant C Remyelination in the CNS: from biology to therapy. Nat Rev Neurosci 2008; 9: 839–55. 18931697 10.1038/nrn2480

[aww039-B41] Fricker FR Antunes-Martins A Galino J Paramsothy R La Russa F Perkins J, Axonal neuregulin 1 is a rate limiting but not essential factor for nerve remyelination. Brain 2013; 136: 2279–97. 23801741 10.1093/brain/awt148 PMC3692042

[aww039-B42] Fricker FR Bennett DL The role of neuregulin-1 in the response to nerve injury. Future Neurol 2011; 6: 809–22. 22121335 10.2217/fnl.11.45 PMC3223410

[aww039-B43] Fricker FR Lago N Balarajah S Tsantoulas C Tanna S Zhu N, Axonally derived neuregulin-1 is required for remyelination and regeneration after nerve injury in adulthood. J Neurosci 2011; 31: 3225–33. 21368034 10.1523/JNEUROSCI.2568-10.2011 PMC3059576

[aww039-B44] Fricker FR Zhu N Tsantoulas C Abrahamsen B Nassar MA Thakur M, Sensory axon-derived neuregulin-1 is required for axoglial signaling and normal sensory function but not for long-term axon maintenance. J Neurosci 2009; 29: 7667–78. Jun 17; 19535578 10.1523/JNEUROSCI.6053-08.2009 PMC2875847

[aww039-B45] Garratt AN Voiculescu O Topilko P Charnay P Birchmeier C A dual role of erbB2 in myelination and in expansion of the schwann cell precursor pool. J Cell Biol 2000; 148: 1035–46. 10704452 10.1083/jcb.148.5.1035 PMC2174554

[aww039-B46] Gaudet AD Popovich PG Extracellular matrix regulation of inflammation in the healthy and injured spinal cord. Exp Neurol 2014; 258: 24–34. 25017885 10.1016/j.expneurol.2013.11.020 PMC4099942

[aww039-B47] Gauthier MK Kosciuczyk K Tapley L Karimi-Abdolrezaee S Dysregulation of the neuregulin-1-ErbB network modulates endogenous oligodendrocyte differentiation and preservation after spinal cord injury. Eur J Neurosci 2013; 38: 2693–715. 23758598 10.1111/ejn.12268

[aww039-B48] Ghatak NR Hirano A Doron Y Zimmerman HM Remyelination in multiple sclerosis with peripheral type myelin. Arch Neurol 1973; 29: 262–7. 4728187 10.1001/archneur.1973.00490280074011

[aww039-B49] Guest JD Hiester ED Bunge RP Demyelination and Schwann cell responses adjacent to injury epicenter cavities following chronic human spinal cord injury. Exp Neurol 2005; 192: 384–93. 15755556 10.1016/j.expneurol.2004.11.033

[aww039-B50] Hagg T Oudega M Degenerative and spontaneous regenerative processes after spinal cord injury. J Neurotrauma 2006; 23: 264–80. 16629615 10.1089/neu.2006.23.263

[aww039-B51] Harrison BM Gledhill RF McDonald WJ Remyelination after transient compression of the spinal cord. Proc Aust Assoc Neurol 1975; 12: 117–22. 1215377

[aww039-B52] Hayashi S McMahon AP Efficient recombination in diverse tissues by a tamoxifen-inducible form of Cre: a tool for temporally regulated gene activation/inactivation in the mouse. Dev Biol 2002; 244: 305–18. 11944939 10.1006/dbio.2002.0597

[aww039-B53] Hippenmeyer S Shneider NA Birchmeier C Burden SJ Jessell TM Arber S A role for neuregulin1 signaling in muscle spindle differentiation. Neuron 2002; 36: 1035–49. 12495620 10.1016/s0896-6273(02)01101-7

[aww039-B54] Hopman AH Ramaekers FC Speel EJ Rapid synthesis of biotin-, digoxigenin-, trinitrophenyl-, and fluorochrome-labeled tyramides and their application for In situ hybridization using CARD amplification. J Histochem Cytochem 1998; 46: 771–7. 9603790 10.1177/002215549804600611

[aww039-B55] Hu X Hicks CW He W Wong P Macklin WB Trapp BD, Bace1 modulates myelination in the central and peripheral nervous system. Nat Neurosci 2006; 9: 1520–5. 17099708 10.1038/nn1797

[aww039-B56] Iaci JF Ganguly A Finklestein SP Parry TJ Ren J Saha S, Glial growth factor 2 promotes functional recovery with treatment initiated up to 7 days after permanent focal ischemic stroke. Neuropharmacology 2010; 59: 640–9. 20691195 10.1016/j.neuropharm.2010.07.017

[aww039-B57] Irvine KA Blakemore WF Remyelination protects axons from demyelination-associated axon degeneration. Brain 2008; 131: 1464–77. 18490361 10.1093/brain/awn080

[aww039-B58] Itoyama Y Ohnishi A Tateishi J Kuroiwa Y Webster HD Spinal cord multiple sclerosis lesions in Japanese patients: Schwann cell remyelination occurs in areas that lack glial fibrillary acidic protein (GFAP). Acta Neuropathol 1985; 65: 217–23. 2579518 10.1007/BF00687001

[aww039-B59] James ND Bartus K Grist J Bennett DL McMahon SB Bradbury EJ Conduction failure following spinal cord injury: functional and anatomical changes from acute to chronic stages. J Neurosci 2011; 31: 18543–55. 22171053 10.1523/JNEUROSCI.4306-11.2011 PMC3495307

[aww039-B60] Jasmin L Janni G Moallem TM Lappi DA Ohara PT Schwann cells are removed from the spinal cord after effecting recovery from paraplegia. J Neurosci 2000; 20: 9215–23. 11124999 10.1523/JNEUROSCI.20-24-09215.2000 PMC6773007

[aww039-B61] Kakulas BA A review of the neuropathology of human spinal cord injury with emphasis on special features. J Spinal Cord Med 1999; 22: 119–24. 10826269 10.1080/10790268.1999.11719557

[aww039-B62] Kanagal SG Muir GD Effects of combined dorsolateral and dorsal funicular lesions on sensorimotor behaviour in rats. Exp Neurol 2008; 214: 229–39. 18778707 10.1016/j.expneurol.2008.08.004

[aww039-B63] Koles ZJ Rasminsky M A computer simulation of conduction in demyelinated nerve fibres. J Physiol 1972; 227: 351–64. 4675037 10.1113/jphysiol.1972.sp010036 PMC1331199

[aww039-B64] Lee Y Morrison BM Li Y Lengacher S Farah MH Hoffman PN, Oligodendroglia metabolically support axons and contribute to neurodegeneration. Nature 2012; 487: 443–8. 22801498 10.1038/nature11314 PMC3408792

[aww039-B65] Li L Cleary S Mandarano MA Long W Birchmeier C Jones FE The breast proto-oncogene, HRGalpha regulates epithelial proliferation and lobuloalveolar development in the mouse mammary gland. Oncogene 2002; 21: 4900–7. 12118369 10.1038/sj.onc.1205634

[aww039-B66] Lovas G Szilagyi N Majtenyi K Palkovits M Komoly S Axonal changes in chronic demyelinated cervical spinal cord plaques. Brain 2000; 123: 308–17. 10648438 10.1093/brain/123.2.308

[aww039-B67] Lundgaard I Luzhynskaya A Stockley JH Wang Z Evans KA Swire M, Neuregulin and BDNF induce a switch to NMDA receptor-dependent myelination by oligodendrocytes. PLoS Biol 2013; 11: e1001743. 24391468 10.1371/journal.pbio.1001743 PMC3876980

[aww039-B68] Makinodan M Rosen KM Ito S Corfas G A critical period for social experience-dependent oligodendrocyte maturation and myelination. Science 2012; 337: 1357–60. 22984073 10.1126/science.1220845 PMC4165613

[aww039-B69] McDonald JW Belegu V Demyelination and remyelination after spinal cord injury. J Neurotrauma 2006; 23: 345–59. 16629621 10.1089/neu.2006.23.345

[aww039-B70] McDonald WI Mechanisms of functional loss and recovery in spinal cord damage. Ciba Found Symp 1975; 34: 23–33. 177252 10.1002/9780470720165.ch3

[aww039-B71] McGavern DB Murray PD Rivera-Quinones C Schmelzer JD Low PA Rodriguez M Axonal loss results in spinal cord atrophy, electrophysiological abnormalities and neurological deficits following demyelination in a chronic inflammatory model of multiple sclerosis. Brain 2000; 123: 519–31. 10686175 10.1093/brain/123.3.519 PMC5444460

[aww039-B72] Mei L Nave KA Neuregulin-ERBB signaling in the nervous system and neuropsychiatric diseases. Neuron 2014; 83: 27–49. 24991953 10.1016/j.neuron.2014.06.007 PMC4189115

[aww039-B73] Meyer D Birchmeier C Multiple essential functions of neuregulin in development. Nature 1995; 378: 386–90. 7477375 10.1038/378386a0

[aww039-B74] Meyer D Yamaai T Garratt A Riethmacher-Sonnenberg E Kane D Theill LE, Isoform-specific expression and function of neuregulin. Development 1997; 124: 3575–86. 9342050 10.1242/dev.124.18.3575

[aww039-B75] Monteiro de Castro G Deja NA Ma D Zhao C Franklin RJ Astrocyte activation via Stat3 signaling determines the balance of oligodendrocyte versus schwann cell remyelination. Am J Pathol 2015; 185: 2431–40. Sep; 26193667 10.1016/j.ajpath.2015.05.011 PMC4597277

[aww039-B76] Nashmi R Fehlings MG Changes in axonal physiology and morphology after chronic compressive injury of the rat thoracic spinal cord. Neuroscience 2001; 104: 235–51. 11311546 10.1016/s0306-4522(01)00009-4

[aww039-B77] Nave KA Salzer JL Axonal regulation of myelination by neuregulin 1. Curr Opin Neurobiol 2006; 16: 492–500. 16962312 10.1016/j.conb.2006.08.008

[aww039-B78] Nave KA Werner HB Myelination of the nervous system: mechanisms and functions. Annu Rev Cell Dev Biol 2014; 30: 503–33. 25288117 10.1146/annurev-cellbio-100913-013101

[aww039-B79] Newbern J Birchmeier C Nrg1/ErbB signaling networks in Schwann cell development and myelination. Semin Cell Dev Biol 2010; 21: 922–8. 20832498 10.1016/j.semcdb.2010.08.008 PMC2991617

[aww039-B80] Norenberg MD Smith J Marcillo A The pathology of human spinal cord injury: defining the problems. J Neurotrauma 2004; 21: 429–40. 15115592 10.1089/089771504323004575

[aww039-B81] Oudega M Bradbury EJ Ramer MS Combination therapies. Handb Clin Neurol 2012; 109: 617–36. 23098740 10.1016/B978-0-444-52137-8.00038-3

[aww039-B82] Papastefanaki F Matsas R From demyelination to remyelination: the road toward therapies for spinal cord injury. Glia 2015; 63: 1101–25. 25731941 10.1002/glia.22809

[aww039-B83] Penderis J Woodruff RH Lakatos A Li WW Dunning MD Zhao C, Increasing local levels of neuregulin (glial growth factor-2) by direct infusion into areas of demyelination does not alter remyelination in the rat CNS. Eur J Neurosci 2003; 18: 2253–64. 14622186 10.1046/j.1460-9568.2003.02969.x

[aww039-B84] Perlin JR Lush ME Stephens WZ Piotrowski T Talbot WS Neuronal Neuregulin 1 type III directs Schwann cell migration. Development 2011; 138: 4639–48. 21965611 10.1242/dev.068072 PMC3190382

[aww039-B85] Plemel JR Keough MB Duncan GJ Sparling JS Yong VW Stys PK, Remyelination after spinal cord injury: is it a target for repair? Prog Neurobiol 2014; 117: 54–72. 24582777 10.1016/j.pneurobio.2014.02.006

[aww039-B86] Potter PJ Disordered control of the urinary bladder after human spinal cord injury: what are the problems? Progr Brain Res 2006; 152: 51–7. 10.1016/S0079-6123(05)52004-1 16198693

[aww039-B87] Raineteau O Schwab ME Plasticity of motor systems after incomplete spinal cord injury. Nat Rev Neurosci 2001; 2: 263–73. 11283749 10.1038/35067570

[aww039-B88] Ramer LM Ramer MS Bradbury EJ Restoring function after spinal cord injury: towards clinical translation of experimental strategies. Lancet Neurol 2014; 13: 1241–56. 25453463 10.1016/S1474-4422(14)70144-9

[aww039-B89] Ravenscroft A Ahmed YS Burnside IG Chronic pain after SCI: a patient survey. Spinal Cord 2000; 38: 611–14. 11093322 10.1038/sj.sc.3101073

[aww039-B90] Salic A Mitchison TJ A chemical method for fast and sensitive detection of DNA synthesis in vivo. Proc Natl Acad Sci USA 2008; 105: 2415–20. 18272492 10.1073/pnas.0712168105 PMC2268151

[aww039-B91] Schwab ME Bartholdi D Degeneration and regeneration of axons in the lesioned spinal cord. Physiol Rev 1996; 76: 319–70. Apr; 8618960 10.1152/physrev.1996.76.2.319

[aww039-B92] Siegenthaler MM Tu MK Keirstead HS The extent of myelin pathology differs following contusion and transection spinal cord injury. J Neurotrauma 2007; 24: 1631–46. 17970626 10.1089/neu.2007.0302

[aww039-B93] Sims TJ Durgun MB Gilmore SA Schwann cell invasion of ventral spinal cord: the effect of irradiation on astrocyte barriers. J Neuropathol Exp Neurol 1998; 57: 866–73. 9737550 10.1097/00005072-199809000-00008

[aww039-B94] Smith KJ Blakemore WF McDonald WI Central remyelination restores secure conduction. Nature 1979; 280: 395–6. 460414 10.1038/280395a0

[aww039-B95] Soderblom C Luo X Blumenthal E Bray E Lyapichev K Ramos J, Perivascular fibroblasts form the fibrotic scar after contusive spinal cord injury. J Neurosci 2013; 33: 13882–7. 23966707 10.1523/JNEUROSCI.2524-13.2013 PMC3755723

[aww039-B96] Stassart RM Fledrich R Velanac V Brinkmann BG Schwab MH Meijer D, A role for Schwann cell-derived neuregulin-1 in remyelination. Nat Neurosci 2013; 16: 48–54. 23222914 10.1038/nn.3281

[aww039-B97] Takeoka A Vollenweider I Courtine G Arber S Muscle spindle feedback directs locomotor recovery and circuit reorganization after spinal cord injury. Cell 2014; 159: 1626–39. 25525880 10.1016/j.cell.2014.11.019

[aww039-B98] Taveggia C Thaker P Petrylak A Caporaso GL Toews A Falls DL, Type III neuregulin-1 promotes oligodendrocyte myelination. Glia 2008; 56: 284–93. 18080294 10.1002/glia.20612

[aww039-B99] Waxman SG Demyelination in spinal cord injury. J Neurol Sci 1989; 91: 1–14. 2664092 10.1016/0022-510x(89)90072-5

[aww039-B100] Waxman SG Utzschneider DA Kocsis JD Enhancement of action potential conduction following demyelination: experimental approaches to restoration of function in multiple sclerosis and spinal cord injury. Prog Brain Res 1994; 100: 233–43. 7938524 10.1016/s0079-6123(08)60790-6

[aww039-B101] Weidner N Tuszynski MH Spontaneous plasticity in the injured spinal cord-implications for repair strategies. Mol Psychiatry 2002; 7: 9–11. 11803438 10.1038/sj.mp.4000983

[aww039-B102] Willem M Garratt AN Novak B Citron M Kaufmann S Rittger A, Control of peripheral nerve myelination by the beta-secretase BACE1. Science 2006; 314: 664–6. 16990514 10.1126/science.1132341

[aww039-B103] Woodruff RH Franklin RJ Demyelination and remyelination of the caudal cerebellar peduncle of adult rats following stereotaxic injections of lysolecithin, ethidium bromide, and complement/anti-galactocerebroside: a comparative study. Glia 1999; 25: 216–28. 9932868 10.1002/(sici)1098-1136(19990201)25:3<216::aid-glia2>3.0.co;2-l

[aww039-B104] Yang X Arber S William C Li L Tanabe Y Jessell TM, Patterning of muscle acetylcholine receptor gene expression in the absence of motor innervation. Neuron 2001; 30: 399–410. 11395002 10.1016/s0896-6273(01)00287-2

[aww039-B105] Zawadzka M Rivers LE Fancy SP Zhao C Tripathi R Jamen F, CNS-resident glial progenitor/stem cells produce Schwann cells as well as oligodendrocytes during repair of CNS demyelination. Cell Stem Cell 2010; 6: 578–90. 20569695 10.1016/j.stem.2010.04.002 PMC3856868

[aww039-B106] Zeng C Pan F Jones LA Lim MM Griffin EA Sheline YI, Evaluation of 5-ethynyl-2'-deoxyuridine staining as a sensitive and reliable method for studying cell proliferation in the adult nervous system. Brain Res 2010; 1319: 21–32. 20064490 10.1016/j.brainres.2009.12.092 PMC2826567

[aww039-B107] Zhang JF Zhao FS Wu G Kong QF Sun B Cao J, Therapeutic effect of co-transplantation of neuregulin-1-transfected Schwann cells and bone marrow stromal cells on spinal cord hemisection syndrome. Neurosci Lett 2011; 497: 128–33. 21539896 10.1016/j.neulet.2011.04.045

