# Supplementary material for: Neuregulin-1 controls an endogenous repair mechanism after spinal cord injury
Source: Brain. 2016 Mar 17;139(5):1394–416. doi: 10.1093/brain/aww039 (PMC5477508; doi:10.1093/brain/aww039)
Supplement: Supplementary Fig. 5 [file suppl_data.zip › brain-2015-01943-File008.pdf]

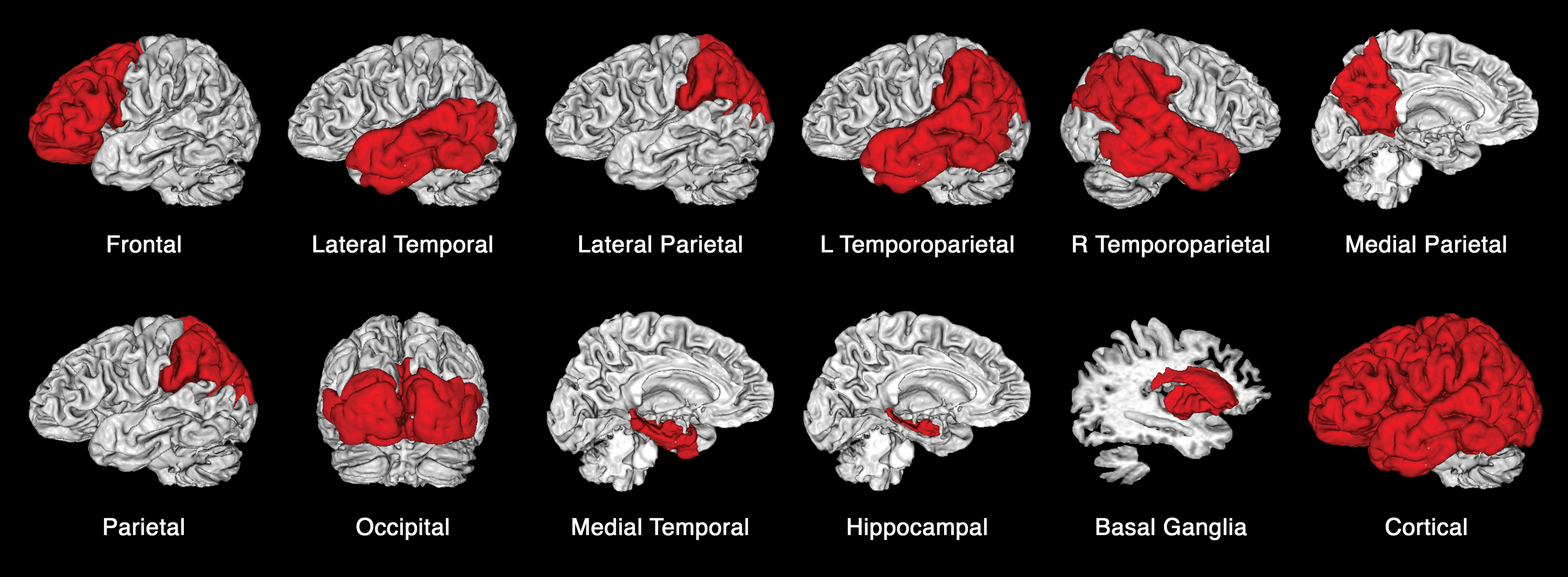
Frontal
Lateral Temporal
Lateral Parietal
L Temporoparietal
R Temporoparietal
Medial Parietal
Parietal
Occipital
Medial Temporal
Hippocampal
Basal Ganglia
Cortical
