# Supplementary material for: Neuregulin-1 controls an endogenous repair mechanism after spinal cord injury
Source: Brain. 2016 Mar 17;139(5):1394–416. doi: 10.1093/brain/aww039 (PMC5477508; doi:10.1093/brain/aww039)
Supplement: Supplementary Fig. 5 [file suppl_data.zip › brain-2015-01943-File010.pdf]

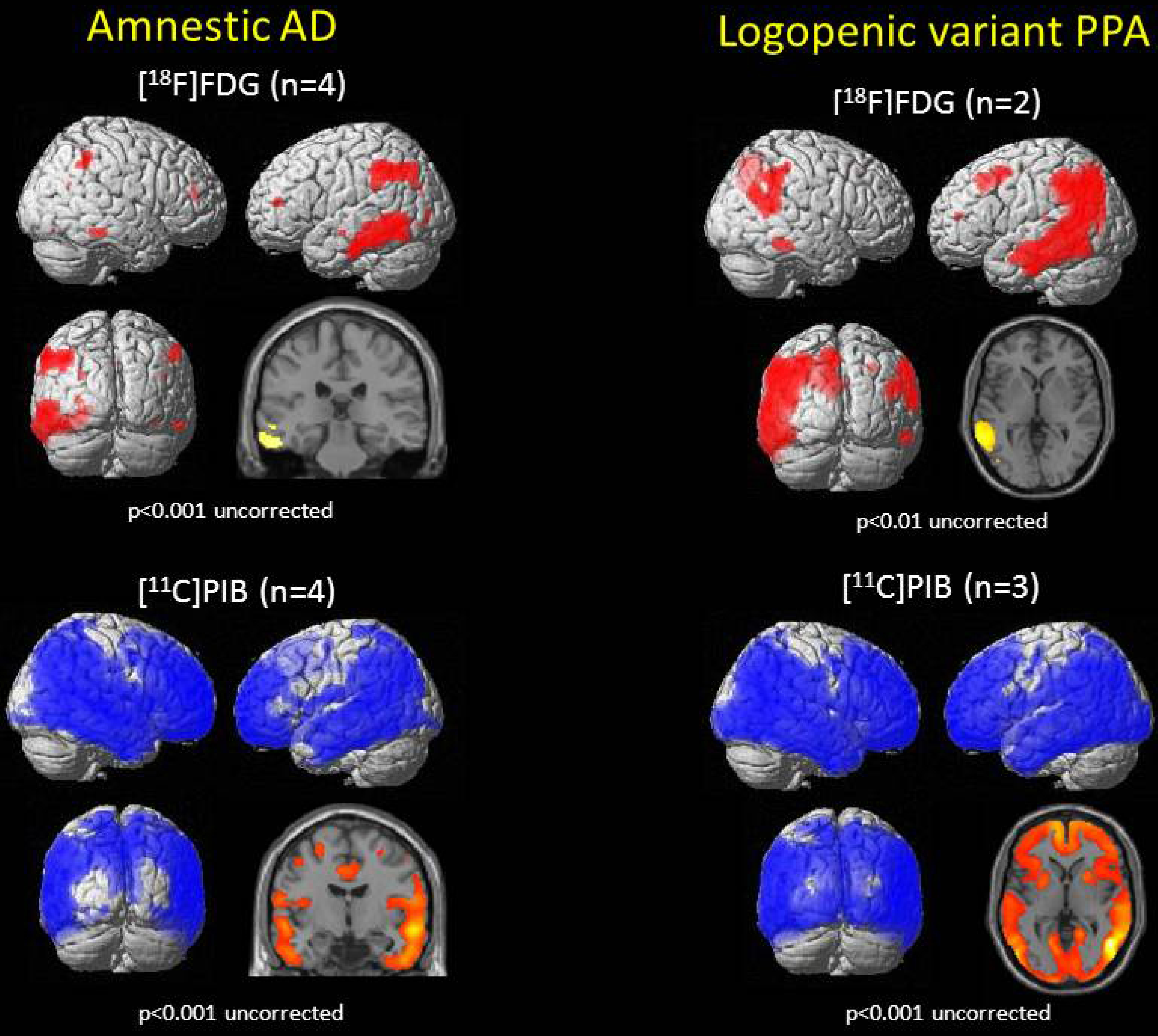
Amnestic AD
Logopenic variant PPA
[18F]FDG (n=4)
p<0.001 uncorrected
[18F]FDG (n=2)
p<0.01 uncorrected
[11C]PIB (n=4)
p<0.001 uncorrected
[11C]PIB (n=3)
p<0.001 uncorrected
