# Supplementary material for: Neuregulin-1 controls an endogenous repair mechanism after spinal cord injury
Source: Brain. 2016 Mar 17;139(5):1394–416. doi: 10.1093/brain/aww039 (PMC5477508; doi:10.1093/brain/aww039)
Supplement: Supplementary Fig. 5 [file suppl_data.zip › brain-2015-01943-File015.pdf]

SUPPLEMENTARY APPENDIX

MRI

Patients underwent MRI, including T1-weighted magnetization prepared rapid gradient echo (MP-RAGE; TR/TE = 2300/2.98ms, FA = 9°, 1x1x1mm resolution) and fluid attenuation inversion recovery (FLAIR) sequences, on a 3-Tesla Siemens Tim Trio scanner at the UCSF Neuroimaging Center. Controls underwent MRI, including T1-weighted magnetization prepared rapid gradient echo (MP-RAGE, TR/TE = 2110/3.58ms, FA = 15°, 1x1x1mm resolution) on 1.5-Tesla Magnetom Avanto scanner at UC Berkeley.
