# Supplementary material for: Neuregulin-1 controls an endogenous repair mechanism after spinal cord injury
Source: Brain. 2016 Mar 17;139(5):1394–416. doi: 10.1093/brain/aww039 (PMC5477508; doi:10.1093/brain/aww039)
Supplement: Supplementary Fig. 5 [file suppl_data.zip › brain-2015-01943-File016.pdf]

FIGURE LEGENDS SUPPLEMENTARY MATERIAL

S-FIGURE 1: Regions-of-interest that were included in the analyses for Figures 3-6 and Supplementary Figures 4-7 are shown on a rendered brain. Each region-of-interest consists of multiple FreeSurfer regions, described in Supplementary Table 1.

S-FIGURE 2: [^{18}F]AV1451 uptake in cognitively normal individuals

Standardized uptake value ratio [^{18}F]AV1451 images (neurological orientation) in the cognitively normal subjects with lowest (left) and highest (right) global [^{18}F]AV1451 uptake of the entire control group. Note that the colorbar is much lower than that of the Alzheimer's disease patients in Figure 1.

S-FIGURE 3: Voxelwise [11C]PIB and [18F]FDG contrasts between amnestic AD and logopenic variant PPA patients against controls

Voxelwise contrasts, thresholded at $p<0.001$ or $p<0.01$ (for [^{18}F]FDG in lvPPA) uncorrected and without covariates, indicating regions in which patients with amnestic Alzheimer's disease and logopenic variant PPA have greater [^{11}C]PIB (blue) and reduced [^{18}F]FDG (red) retention compared to healthy controls.

S-FIGURE 4*: Partial volume corrected [^{18}F]AV1451 uptake and hemispheric asymmetry in regions-of-interest

Panel A shows partial volume corrected [^{18}F]AV1451 SUVR values for each AD patient and controls in seven bilateral regions-of-interest. Panel B shows the degree of asymmetric tracer uptake within each ROI as the percentage difference in SUVR value for left compared to right hemisphere: asymmetry index [%] = $200 \times (R - L) / (R + L)$. Table 2 and

Supplementary Table 2 shows the group means and differences of [^{18}F]AV1451 standardized uptake value ratios and asymmetry indices.

** Remake of Figure 3 but now includes partial volume corrected data*

S-FIGURE 5*. Direct comparisons between PET tracers in partial volume corrected region-of-interest

Across 16 Alzheimer's disease patients, regions with greater [^{18}F]AV1451 uptake were strongly associated with lower [^{18}F]FDG metabolism (A) and between [^{18}F]AV1451 and [^{11}C]PIB (C). There was no relationship between [^{11}C]PIB and [^{18}F]FDG (B).

** Remake of Figure 4 but now includes partial volume corrected data*

S-FIGURE 6*. Patterns of [^{18}F]AV1451 retention in Alzheimer's disease associated with age, Apolipoprotein E ε4 status and cognitive performance

Results from voxelwise linear regression are displayed at $p<0.01$ uncorrected for multiple comparisons, without covariates for age (A), adjusted for global amyloid-β burden for Apolipoprotein E ε4 status (B), and without covariates for memory (C), visuospatial (D), and language testing (E).

** Remake of the voxelwise analyses shown in Figure 5 and 6 but now at a slightly more stringent threshold (p<0.01 uncorrected)*

S-FIGURE 7*. Partial volume corrected associations between regional [^{18}F]AV1451, age and cognitive performance

Partial volume corrected region-of-interest analyses demonstrate a positive correlation between increasing age and hippocampal-to-cortical [^{18}F]AV1451 (A) and show that

cognitive impairment was associated with focal rather than global increases in $[^{18}F]$AV1451 in regions associated with memory (B), visuospatial (C), and language testing (D).

** Remake of the region-of-interest analyses shown in Figures 5 and 6 but now including partial volume corrected data*
