# Supplementary material for: Neuregulin-1 controls an endogenous repair mechanism after spinal cord injury
Source: Brain. 2016 Mar 17;139(5):1394–416. doi: 10.1093/brain/aww039 (PMC5477508; doi:10.1093/brain/aww039)
Supplement: Supplementary Fig. 5 [file suppl_data.zip › brain-2015-01943-File017.pdf]

Supplementary Table 1. FreeSurfer ROIs, lables and names

Combined Region	FreeSurfer Labels	FreeSurfer Label Names	Fig 3	Fig 4	Fig 5	Fig 6
Frontal	1003, 1012, 1014, 1018, 1019, 1020, 1027, 1028, 1032, 2003, 2012, 2014, 2018, 2019, 2020, 2027, 2028, 2032	ctx-lh-caudalmiddlefrontal, ctx-lh-lateralorbitofrontal, ctx-lh-medialorbitofrontal, ctx-lh-parsopercularis, ctx-lh-parsorbitalis, ctx-lh-parstriangularis, ctx-lh-rostralmiddlefrontal, ctx-lh-superiorfrontal, ctx-lh-frontalpole, ctx-rh-caudalmiddlefrontal, ctx-rh-lateralorbitofrontal, ctx-rh-medialorbitofrontal, ctx-rh-parsopercularis, ctx-rh-parsorbitalis, ctx-rh-parstriangularis, ctx-rh-rostralmiddlefrontal, ctx-rh-superiorfrontal, ctx-rh-frontalpole	x	x		
Lateral Temporal	1001, 1009, 1015, 1030, 1034, 2001, 2009, 2015, 2030, 2034	ctx-lh-bankssts, ctx-lh-inferiortemporal, ctx-lh-middletemporal, ctx-lh-superiortemporal, ctx-lh-transversetemporal, ctx-rh-bankssts, ctx-rh-inferiortemporal, ctx-rh-middletemporal, ctx-rh-superiortemporal, ctx-rh-transversetemporal	x	x		
Lateral Parietal	1008, 1029, 1031, 2008, 2029, 2031	ctx-lh-inferiorparietal, ctx-lh-superiorparietal, ctx-lh-supramarginal, ctx-rh-inferiorparietal, ctx-rh-superiorparietal, ctx-rh-supramarginal	x			
L Temporoparietal	1001, 1008, 1009, 1015, 1030, 1031,	ctx-lh-bankssts, ctx-lh-inferiorparietal, ctx-lh-inferiortemporal,				x

	1034	ctx-lh-middletemporal, ctx-lh-superiortemporal, ctx-lh-supramarginal, ctx-lh-transversetemporal				
R Temporoparietal	2001, 2008, 2009, 2015, 2030, 2031, 2034	ctx-rh-bankssts, ctx-rh-inferiorparietal, ctx-rh-inferiortemporal, ctx-rh-middletemporal, ctx-rh-superiortemporal, ctx-rh-supramarginal, ctx-rh-transversetemporal				x
Medial Parietal	1010, 1025, 2010, 2025	ctx-lh-isthmuscingulate, ctx-lh-precuneus, ctx-rh-isthmuscingulate, ctx-rh-precuneus	x			
Parietal	1008, 1025, 1029, 1031, 2008, 2025, 2029, 2031	ctx-lh-inferiorparietal, ctx-lh-precuneus, ctx-lh-superiorparietal, ctx-lh-supramarginal, ctx-rh-inferiorparietal, ctx-rh-precuneus, ctx-rh-superiorparietal, ctx-rh-supramarginal		x		
Occipital	1005, 1011, 1013, 1021, 2005, 2011, 2013, 2021	ctx-lh-cuneus, ctx-lh-lateraloccipital, ctx-lh-lingual, ctx-lh-pericalcarine, ctx-rh-cuneus, ctx-rh-lateraloccipital, ctx-rh-lingual, ctx-rh-pericalcarine	x	x		x
Medial Temporal	17, 53, 1006, 1016, 2006, 2016	Left-Hippocampus, Right-Hippocampus, ctx-lh-entorhinal, ctx-lh-parahippocampal, ctx-rh-entorhinal, ctx-rh-parahippocampal	x			
Hippocampal	17, 53	Left-Hippocampus, Right-Hippocampus			x	x
Basal Ganglia	11, 12, 13, 50, 51, 52	Left-Caudate, Left-Putamen, Left-Pallidum, Right-Caudate, Right-Putamen, Right-Pallidum	x			

Cortical	17, 53, 1001, 1002, 1003, 1005, 1006, 1007, 1008, 1009, 1010, 1011, 1012, 1013, 1014, 1015, 1016, 1017, 1018, 1019, 1020, 1021, 1022, 1023, 1024, 1025, 1026, 1027, 1028, 1029, 1030, 1031, 1032, 1033, 1034, 1035, 2001, 2002, 2003, 2005, 2006, 2007, 2008, 2009, 2010, 2011, 2012, 2013, 2014, 2015, 2016, 2017, 2018, 2019, 2020, 2021, 2022, 2023, 2024, 2025, 2026, 2027, 2028, 2029, 2030, 2031, 2032, 2033, 2034, 2035	Left-Hippocampus, Right-Hippocampus, ctx-lh-bankssts, ctx-lh-caudalanteriorcingulate, ctx-lh-caudalmiddlefrontal, ctx-lh-cuneus, ctx-lh-entorhinal, ctx-lh-fusiform, ctx-lh-inferiorparietal, ctx-lh-inferiortemporal, ctx-lh-isthmuscingulate, ctx-lh-lateraloccipital, ctx-lh-lateralorbitofrontal, ctx-lh-lingual, ctx-lh-medialorbitofrontal, ctx-lh-middletemporal, ctx-lh-parahippocampal, ctx-lh-paracentral, ctx-lh-parsopercularis, ctx-lh-parsorbitalis, ctx-lh-parstriangularis, ctx-lh-pericalcarine, ctx-lh-postcentral, ctx-lh-posteriorcingulate, ctx-lh-precentral, ctx-lh-precuneus, ctx-lh-rostralanteriorcingulate, ctx-lh-rostralmiddlefrontal, ctx-lh-superiorfrontal, ctx-lh-superiorparietal, ctx-lh-superiortemporal, ctx-lh-supramarginal, ctx-lh-frontalpole, ctx-lh-temporalpole, ctx-lh-transversetemporal, ctx-lh-insula, ctx-rh-bankssts, ctx-rh-caudalanteriorcingulate, ctx-rh-caudalmiddlefrontal, ctx-rh-cuneus, ctx-rh-entorhinal, ctx-rh-fusiform, ctx-rh-inferiorparietal, ctx-rh-inferiortemporal, ctx-rh-isthmuscingulate, ctx-rh-lateraloccipital, ctx-rh-lateralorbitofrontal, ctx-rh-lingual, ctx-rh-medialorbitofrontal,			x	x

		ctx-rh-middletemporal, ctx-rh-parahippocampal, ctx-rh-paracentral, ctx-rh-parsopercularis, ctx-rh-parsorbitalis, ctx-rh-parstriangularis, ctx-rh-pericalcarine, ctx-rh-postcentral, ctx-rh-posteriorcingulate, ctx-rh-precentral, ctx-rh-precuneus, ctx-rh-rostralanteriorcingulate, ctx-rh-rostralmiddlefrontal, ctx-rh-superiorfrontal, ctx-rh-superiorparietal, ctx-rh-superiortemporal, ctx-rh-supramarginal, ctx-rh-frontalpole, ctx-rh-temporalpole, ctx-rh-transversetemporal, ctx-rh-insula				

Supplementary Table 2. [^{18}F]AV1451, [^{18}F]FDG and [^{11}C]PIB retention values and asymmetry indices

	**Non-amnestic AD** n=1		**Behavioral AD** n=1		**Corticobasal syndrome** n=1	
**[^{18}F]AV1451**	*SUVR*	*AI (%)*	*SUVR*	*AI (%)*	*SUVR*	*AI (%)*
Frontal	1.45	-1.9	1.27	-1.4	1.51	22.0
Lateral temporal	1.88	-3.9	1.40	-8.3	2.4	15.5
Lateral parietal	2.29	-1.3	1.61	-8.2	2.36	7.2
Medial parietal	2.32	7.7	1.71	-0.9	1.96	27.7
Occipital	1.71	-2.9	1.42	-11.4	1.79	24.7
Medial Temporal	1.40	-5.4	1.23	-7.4	1.69	5.3
Basal ganglia	1.27	0.1	1.32	-3.6	1.28	4.7
**[^{18}F]FDG**	*SUVR*	*AI (%)*	*SUVR*	*AI (%)*	*SUVR*	*AI (%)*
Frontal	1.61	-1.2	1.59	-3.2	1.71	11.0
Lateral temporal	1.28	-2.6	1.27	-10.3	1.13	30.8
Lateral parietal	1.26	-3.0	1.25	-6.1	1.11	36.3
Medial parietal	1.61	0.3	1.45	1.6	1.52	28.2
Occipital	1.77	-4.6	1.62	-5.0	1.55	24.9
Medial Temporal	1.17	-2.8	1.06	-7.2	1.08	25.0
Basal ganglia	1.57	-4.4	1.37	-6.2	1.67	16.7

**[^{11}C]PIB**	*DVR*	*AI (%)*	*DVR*	*AI (%)*	*DVR*	*AI (%)*
Frontal	1.77	2.7	1.96	0.7	1.63	-1.2
Lateral temporal	1.65	-0.2	1.75	1.5	1.40	-6.2
Lateral parietal	1.71	2.2	1.79	-3.1	1.53	-11.6
Medial parietal	1.89	3.1	2.12	-6.3	1.77	-1.0
Occipital	1.36	2.7	1.62	-2.6	1.40	2.7
Medial Temporal	1.21	2.0	1.14	4.2	0.98	-9.0
Basal ganglia	1.65	3.3	1.53	2.7	1.35	-4.6

Asymmetry indices (AI) for [^{18}F]AV1451 and [^{11}C]PIB were calculated using AI [%] = 200 × (R − L) / (R + L), and AI [%] = -200 × (R − L) / (R + L) for [^{18}F]FDG. See Figure 3 for individual data.

AD = Alzheimer's disease; SUVR = Standardized uptake value ratio; AI = Asymmetry index DVR = Distribution volume ratio.

Supplementary Table 3. Relationships between PET tracers within cortical regions

	No partial volume correction				Partial volume corrected			
**Model 1: AV1451 → FDG**	**Beta**	**SE**	**X^2**	**p**	**Beta**	**SE**	**X^2**	**p**
Frontal	-0.175	0.029	30.916	<0.001	-0.144	0.026	27.004	<0.001
Lateral_temporal	-0.303	0.037	38.065	<0.001	-0.124	0.027	17.338	<0.001
Parietal	-0.197	0.030	34.567	<0.001	-0.150	0.021	36.461	<0.001
Occipital	-0.303	0.047	30.896	<0.001	-0.127	0.039	8.520	0.004
**Model 2: PIB → FDG**								
Frontal	0.347	0.084	15.7	<0.001	0.152	0.074	3.615	0.057
Lateral temporal	0.237	0.096	5.925	0.015	0.065	0.056	1.240	0.266
Parietal	0.56	0.089	34.202	<0.001	0.114	0.052	4.518	0.034
Occipital	0.11	0.136	0.644	0.422	-0.051	0.098	0.266	0.606
**Model 3: PIB → AV1451**								
Frontal	0.283	0.276	1.044	0.307	0.796	0.263	8.289	0.004

Lateral_temporal	-0.13	0.309	0.167	0.682		0.970	0.210	19.037	<0.001
Parietal	0.723	0.220	10.443	0.001		1.286	0.166	52.135	<0.001
Occipital	0.753	0.237	9.466	0.002		1.026	0.208	21.626	<0.001

In addition to an overall model including all regions-of-interest (Figure 4 and Supplementary Figure 5), we also performed mixed effects models within four distinct cortical regions for both partial volume corrected and uncorrected data (model 1: AV1451 predicting FDG, model 2: PIB predicting FDG, and model 3: PIB predicting AV1451). Likelihood ratio tests (X^2) were used to assess significance.
