# Supplementary figures and images for: Neuregulin-1 controls an endogenous repair mechanism after spinal cord injury
Source: Brain. 2016 Mar 17;139(5):1394–416. doi: 10.1093/brain/aww039 (PMC5477508; doi:10.1093/brain/aww039)

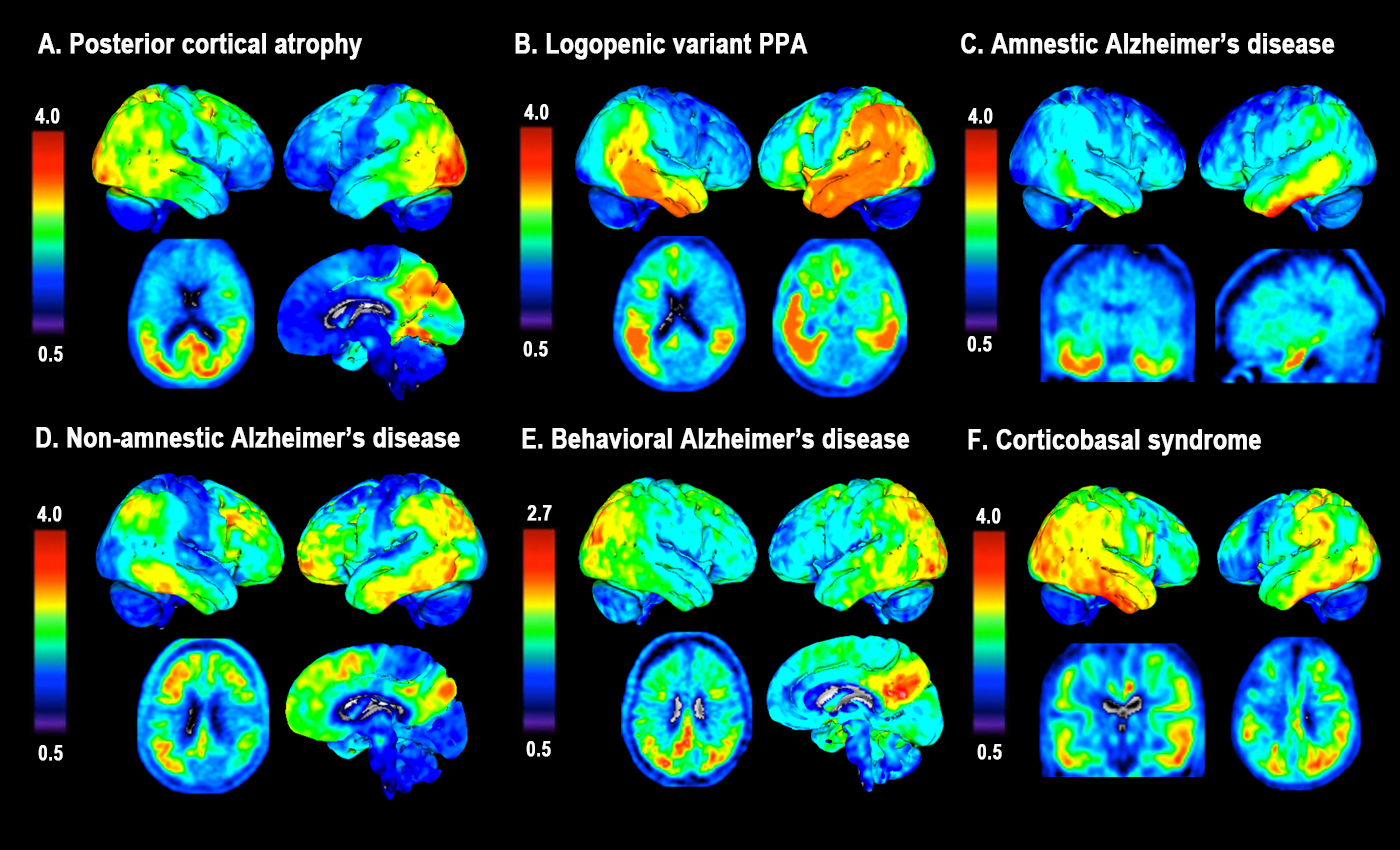

Supplement: Supplementary Fig. 5 [file suppl_data.zip › brain-2015-01943-File019.jpg]

**Healthy control #1**

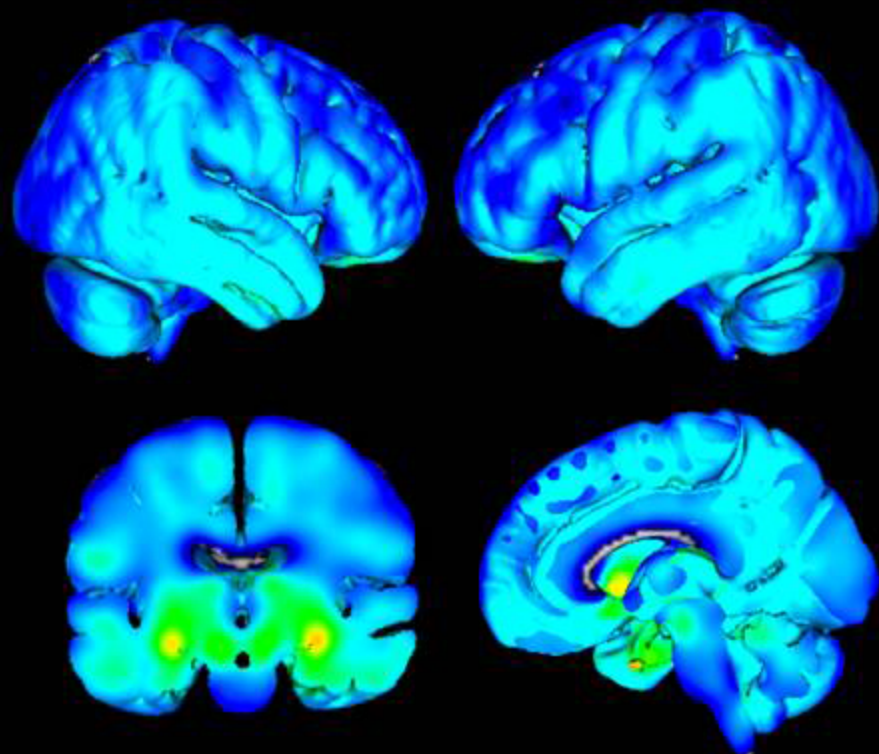

0.6

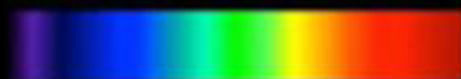

2.2

AV1451 SUVR

**Healthy control #2**

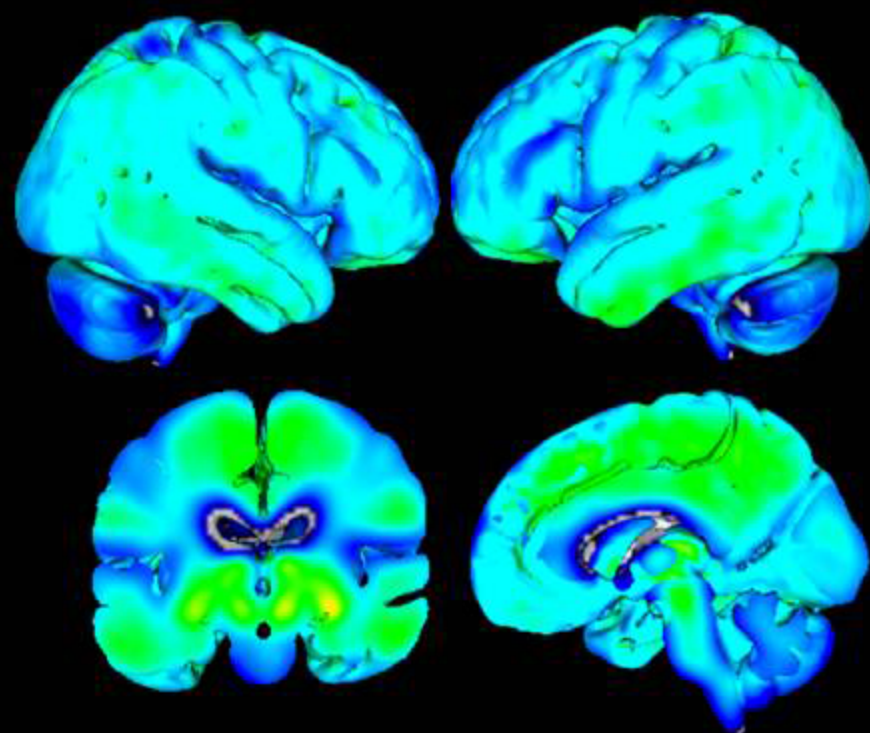

0.6

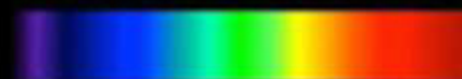

2.2

AV1451 SUVR

Supplement: Supplementary Fig. 5 [file suppl_data.zip › brain-2015-01943-File009.pdf]

AV1451 Mean SUVR (PVC)

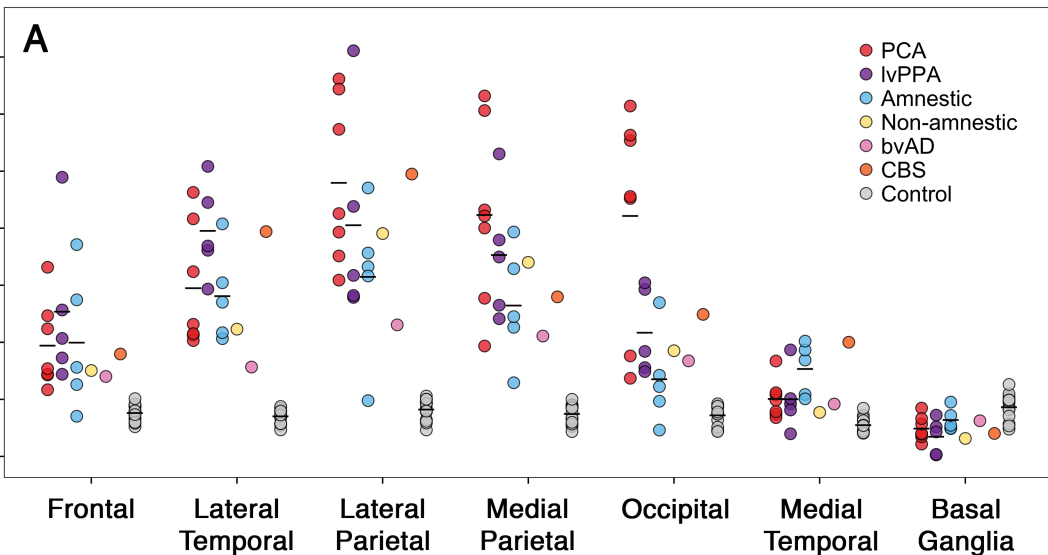

AV1451 Asymmetry Index (%)

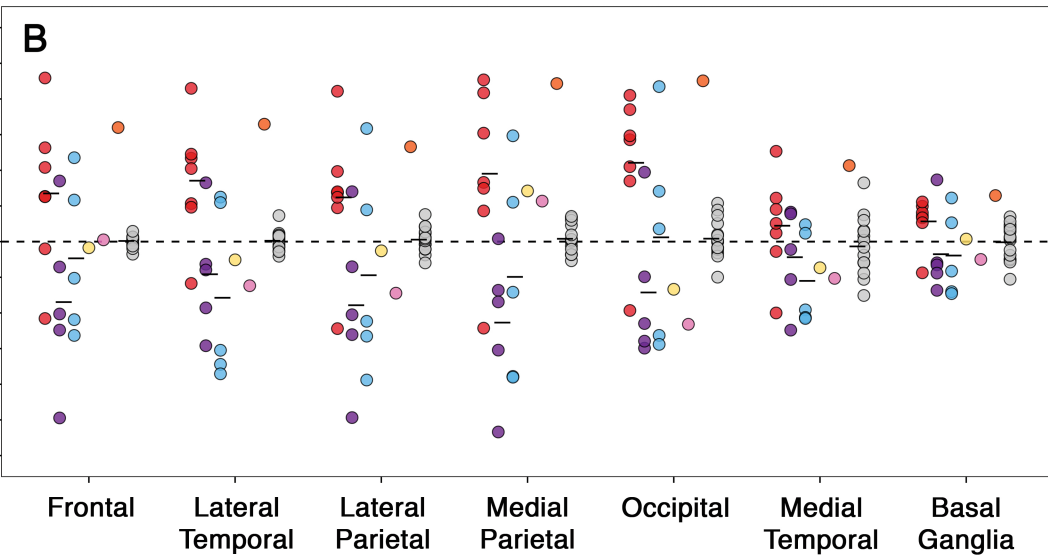

Supplement: Supplementary Fig. 5 [file suppl_data.zip › brain-2015-01943-File011.pdf]

**A.**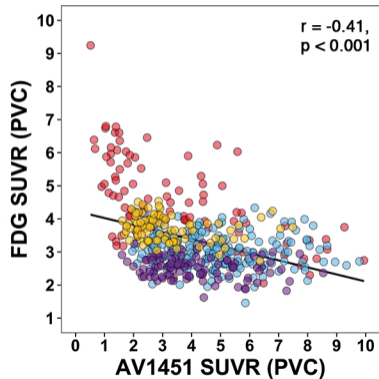**B.**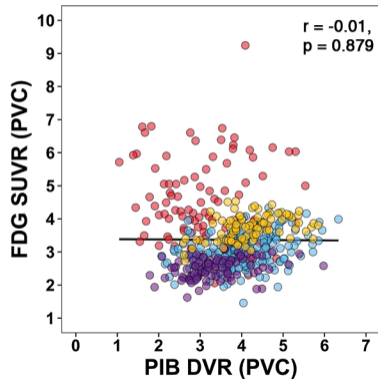**C.**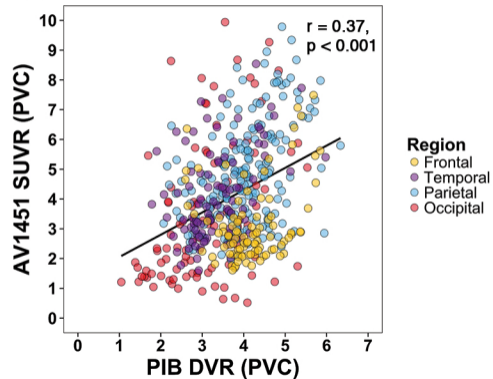

Supplement: Supplementary Fig. 5 [file suppl_data.zip › brain-2015-01943-File012.pdf]

## A. Age

## B. APOE

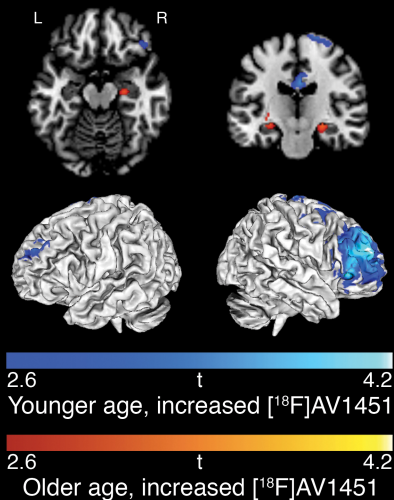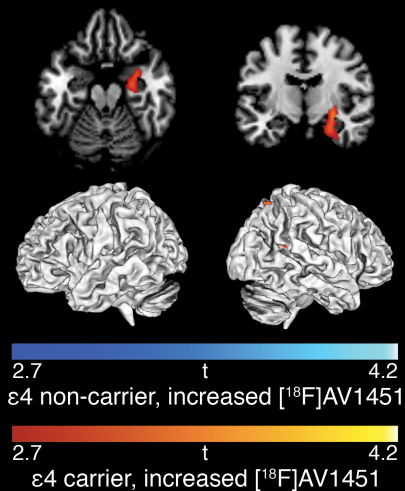

## C. Memory

## D. Visuospatial

## E. Language

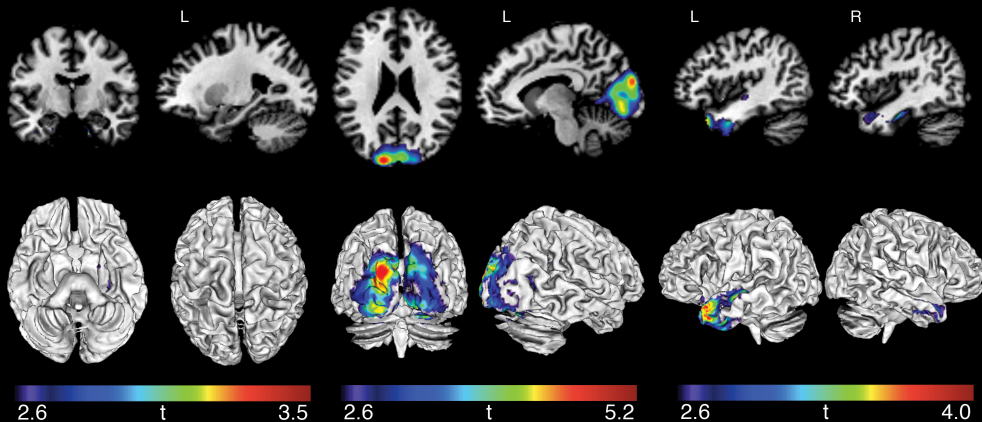

Supplement: Supplementary Fig. 5 [file suppl_data.zip › brain-2015-01943-File013.pdf]

## A. Age

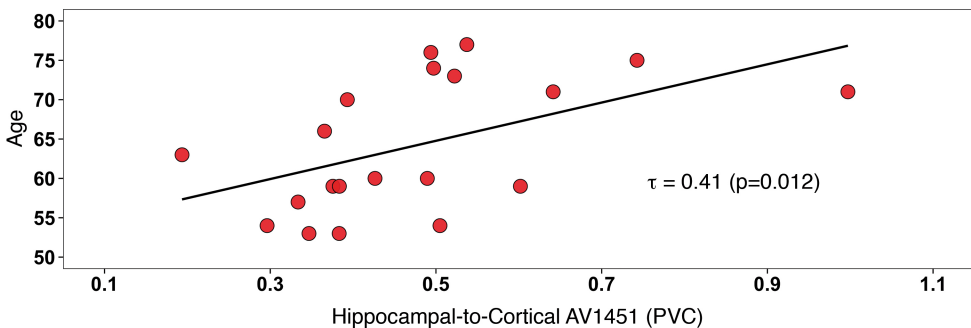

## B. Memory

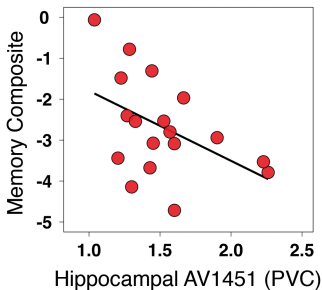

## C. Visuospatial

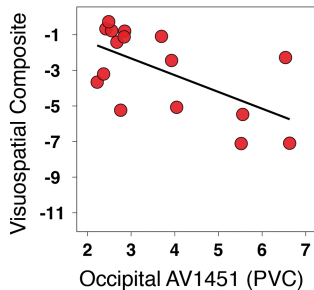

## D. Language

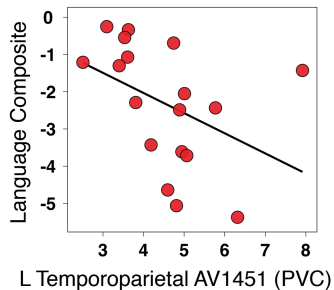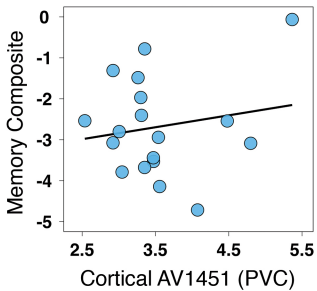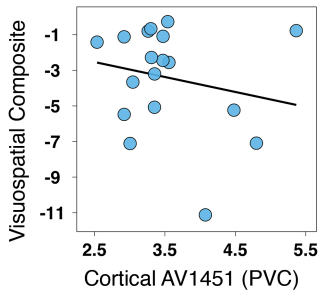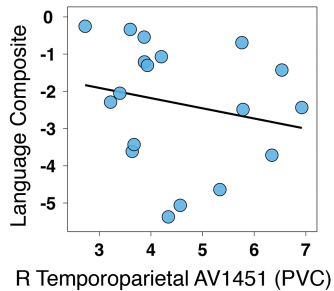

Supplement: Supplementary Fig. 5 [file suppl_data.zip › brain-2015-01943-File014.pdf]
